# The dual ubiquitin binding mode of SPRTN secures rapid spatiotemporal proteolysis of DNA–protein crosslinks

**DOI:** 10.1093/nar/gkaf638

**Published:** 2025-07-21

**Authors:** Wei Song, Yichen Zhao, Annamaria Ruggiano, Christina Redfield, Joseph A Newman, Xiaosheng Zhu, Marta García-Flores, Abimael Cruz-Migoni, Rebecca Roddan, Anna Pérez-Ràfols, Peter J McHugh, Paul R Elliott, Kristijan Ramadan

**Affiliations:** Department of Oncology, MRC Weatherall Institute of Molecular Medicine, John Radcliffe Hospital, University of Oxford, Oxford, OX3 9DS, UK; Department of Oncology, MRC Weatherall Institute of Molecular Medicine, John Radcliffe Hospital, University of Oxford, Oxford, OX3 9DS, UK; Department of Oncology, MRC Weatherall Institute of Molecular Medicine, John Radcliffe Hospital, University of Oxford, Oxford, OX3 9DS, UK; Center for Biological Research Margarita Salas (CIB-CSIC), Spanish National Research Council, Madrid, 28040, Spain; Department of Biochemistry, University of Oxford, Oxford, OX1 3QU, UK; Centre for Medicines Discovery, University of Oxford, Oxford, OX3 7FZ, UK; Department of Oncology, MRC Weatherall Institute of Molecular Medicine, John Radcliffe Hospital, University of Oxford, Oxford, OX3 9DS, UK; Center for Biological Research Margarita Salas (CIB-CSIC), Spanish National Research Council, Madrid, 28040, Spain; Department of Oncology, MRC Weatherall Institute of Molecular Medicine, John Radcliffe Hospital, University of Oxford, Oxford, OX3 9DS, UK; Department of Oncology, MRC Weatherall Institute of Molecular Medicine, John Radcliffe Hospital, University of Oxford, Oxford, OX3 9DS, UK; MRC Protein Phosphorylation and Ubiquitylation Unit, Sir James Black Centre, School of Life Sciences, University of Dundee, Dundee, DD1 5EH, UK; Department of Oncology, MRC Weatherall Institute of Molecular Medicine, John Radcliffe Hospital, University of Oxford, Oxford, OX3 9DS, UK; Department of Biochemistry, University of Oxford, Oxford, OX1 3QU, UK; Department of Oncology, MRC Weatherall Institute of Molecular Medicine, John Radcliffe Hospital, University of Oxford, Oxford, OX3 9DS, UK; Lee Kong Chian School of Medicine (LKCMedicine), Cancer Discovery and Regenerative Medicine Program, Nanyang Technological University, 636921, Singapore

## Abstract

DNA–protein crosslinks (DPCs) are endogenous and chemotherapy-induced genotoxic DNA lesions and, if not repaired, lead to embryonic lethality, neurodegeneration, premature ageing, and cancer. DPCs are heavily polyubiquitinated, and the SPRTN protease and 26S proteasome emerged as two central enzymes for DPC proteolysis. The proteasome recognizes its substrates by their ubiquitination status. How SPRTN protease, an essential enzyme for DPC proteolysis, achieves specificity for DPCs is still not entirely clear. We found that the N-terminal SPRTN catalytic region (SprT) possesses a ubiquitin-binding domain that we named the Ubiquitin Interface of SprT Domain (USD). Using multiple biochemical, biophysical, and structural approaches, we reveal that USD binds ubiquitin chains in an avidity manner. SPRTN binding to ubiquitin chains via USD leads to ∼67-fold higher activation of SPRTN proteolysis towards polyubiquitinated DPCs than the unmodified DPCs. In contrast, the constitutive components of the replisome during unperturbed or translesional DNA synthesis, namely proliferating cell nuclear antigen (PCNA) or monoUb-PCNA, respectively, were poorly degraded, if at all, by SPRTN. This study reveals that the poly-ubiquitination of DPCs serves as the key signal for SPRTN’s rapid proteolysis and determines its substrate specificity towards DPCs, rather than the replisome.

## Graphical Abstract

**Graphical Abstract:**
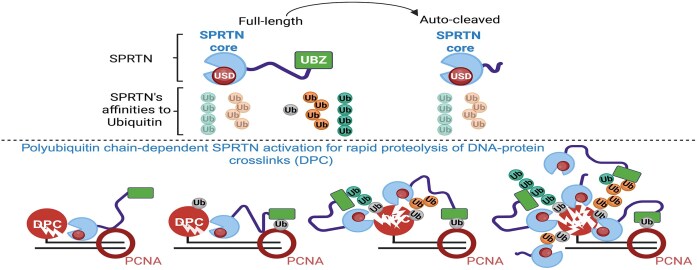

## Introduction

DNA–protein crosslinks (DPCs) are bulky cytotoxic DNA lesions induced by the covalent attachment of chromatin-associated proteins to DNA in the presence of crosslinking agents [1–7]. DPCs form with exposure to endogenous metabolic products, such as aldehydes and chemotherapy drugs, such as topoisomerase inhibitors and oxaliplatin. DPCs can also form due to the physiological or abortive actions of DNA metabolic enzymes. Examples include the binding of HMCES (5hmC-binding, embryonic stem-cell-specific protein) to sites of abasic lesions, which protects single-stranded DNA from breaks during replication, and the covalent trapping of topoisomerases at endogenous DNA lesions [8–10]. DPCs disrupt chromatin structures and suppress the progression of DNA replication and transcription due to their bulkiness. If not repaired, DPCs lead to embryonic lethality, premature ageing, neurodegeneration, and cancer [11–18]. Therefore, understanding the DPC repair mechanisms is essential for preventing these diseases and improving cancer therapies.

The study of DPC repair has been significantly boosted over the last decade by discovering a specialized replication-coupled DPC proteolysis repair pathway in yeast and vertebrates [11, 19–23]. During DNA replication, the mammalian DNA metalloprotease SPRTN (also known as DVC1) interacts with the replisome and travels alongside DNA replication [19, 24–26]. As DNA-dependent proteases, SPRTN and its yeast counterpart, Wss1, cleave the protein component of the DPC, leaving a remnant peptide that enables the progression of the DNA replication fork via translesion synthesis DNA polymerases [20, 27]. Notably, SPRTN knock-out causes early embryonic lethality in mice, and its biallelic mutations or down-regulation cause Ruijs-Aalfs syndrome (RJALS) in humans or RJALS-like phenotype in mice, respectively [11, 12]. RJALS is characterized by chromosomal instability, accelerated ageing/segmental progeria, and early-onset hepatocellular carcinoma [11, 12]. SPRTN is a DNA-dependent metalloprotease but lacks substrate specificity [3, 19, 22, 23]. Therefore, SPRTN must be tightly regulated to avoid unintended cleavage of the DNA replication machinery or nonspecific proteolysis of functional chromatin proteins [28].

In recent years, several levels of SPRTN regulation have been discovered [29, 30]. SPRTN’s activity strictly depends on DNA binding. This is achieved through two DNA-binding interfaces, the Zn-binding domain (ZBD), and the basic DNA-binding region (BR) in the N-terminal part of SPRTN, close to the active centre of its protease activity [3, 31]. SPRTN’s activity is highly unselective *in vitro*, where virtually any DNA-bound protein can be proteolysed, including SPRTN itself [19, 22, 23]. This autocleavage activity is deemed to be a mechanism of self-inactivation [22, 32]. Whether SPRTN targets substrates or undergoes autocleavage depends on the type of activating DNA, although controversies exist. Some studies have demonstrated that short single-stranded oligonucleotides (60mer) and circular single-stranded (ss) DNA plasmids induce efficient SPRTN protease activity towards substrates [22]. In contrast, double-stranded (ds) DNA mainly governs its activity for auto-cleavage [22]. Other studies claim that dsDNA also activates SPRTN towards substrates [19, 23]. Regardless, the best activating DNA structure *in vitro* is the ss/dsDNA junction (dsDNA with overhang) [3]. During DNA replication, a similar DNA structure would result from uncoupling between the DPC-stalled DNA polymerase and the CMG helicase, which might bypass the DPC lesion [33].

The second level of SPRTN regulation relies on ubiquitination, but the mechanism is largely elusive and controversial. SPRTN possesses a Ub-binding Zn-finger (UBZ) domain that directly binds to ubiquitin. Data from *in vitro* set-up and a cell-free system suggest that SPRTN processing of substrates occurs independently of their ubiquitination [3, 19, 22, 23, 27, 31]. A partial explanation is that the SPRTN UBZ domain may not directly bind to DPC substrates; instead, it may engage with other ubiquitinated proteins in the vicinity of DPCs [27]. In addition, the recruitment of SPRTN to formaldehyde-induced nuclear repair foci relies on a viable ubiquitination signal and a functional UBZ domain [34, 35]. One candidate is PCNA, a ubiquitination target in response to DNA damage [36]. This leads to a widely accepted dogma in the field: SPRTN is involved in the processing of DPCs independently of their ubiquitination status, but its specificity towards DPCs is regulated by the ss/dsDNA structure [27]. Additionally, poly-ubiquitinated DPCs are targeted for 26S proteasome recruitment and processing [8, 29, 37–39]. It has been reported that SPRTN is activated to process HMCES by nascent DNA synthesis up to the lesion [40]. In parallel, HMCES is ubiquitinated by RPA/ssDNA binding E3 ligase RFWD3 and consequently degraded. However, whether polyubiquitination on HMCES at abasic sites also activates SPRTN proteolysis or polyubiquitinated HMCES is solely degraded by the proteasome is not clear. Interestingly, recent data on a defined plasmid-based DPC analysed in Xenopus egg extracts demonstrate that SPRTN degrades HMCES-DPC localized at abasic sites behind the DNA replication fork. Moreover, while HMCES is polyubiquitinated, proteasome inhibition does not stabilize HMCES on abasic sites [40]. This suggests a more specific role for SPRTN in processing ubiquitinated DPCs than the proteasome, at least in the context of post-replicative DNA synthesis. Altogether, this indicates that polyubiquitination on DPCs plays a role in SPRTN regulation.

Ubiquitin also seems to regulate SPRTN protease activity. It has been reported that adding ubiquitin to the reaction *in vitro* facilitates SPRTN autocleavage and proteolysis towards Top2α, but there is no further understanding of this effect [23]. Recent data suggest that monoubiquitination on SPRTN is a regulatory mechanism for SPRTN inactivation and consequent degradation by autocleavage and proteasomal degradation [32].

Altogether, varying evidence suggests that the regulation of SPRTN recruitment and proteolysis of DPCs depends on ubiquitin and ubiquitination at/around DNA damage sites. It is currently clear that the main activator of SPRTN proteolysis is the ss/dsDNA structure, which is formed during DNA replication fork progression or post-replicative DNA gap synthesis when nascent DNA approaches DPCs. However, the exact role of ubiquitinated DPCs in SPRTN proteolysis has yet to be characterized and understood.

We hypothesized that ubiquitin and the ubiquitin signal have a much more direct role in regulating SPRTN-dependent DPC proteolysis and might contribute to SPRTN’s specificity towards substrates.

To test our hypothesis, we first engineered a high-sensitivity fluorescence-based assay to monitor SPRTN proteolysis in combination with ubiquitin *in vitro*. Using biophysical methods to address protein–protein interactions, including isothermal titration calorimetry (ITC) and NMR, we discovered that SPRTN has dual ubiquitin-binding properties that govern its rapid proteolysis of polyubiquitinated substrates, including DPCs. Specifically, the C-terminal UBZ domain of SPRTN, which has a high affinity to ubiquitin, acts as a general sensor for ubiquitinated substrates. In contrast, the here identified N-terminal ubiquitin-binding interface (USD), located on the SprT domain, allows SPRTN to selectively bind to polyubiquitinated substrates in an avidity manner and, consequently, facilitates their rapid proteolysis. The polyubiquitin chains on DPCs potentiate the enzymatic activity of SPRTN, making the substrate processing remarkably faster than non-ubiquitinated substrates. In parallel, SPRTN barely cleaves PCNA or monoUb-PCNA, suggesting that components of the replisome that are not polyubiquitinated are protected from SPRTN degradation. Our work has resolved a long-standing question in the field—namely, how SPRTN gains its substrate/DPC specificity while simultaneously not cleaving the replisome. In conclusion, we demonstrate that polyubiquitin chains on DPCs are essential catalysts for SPRTN, revealing an exquisitely fine-tuned regulation of SPRTN proteolysis towards polyubiquitinated DPCs.

## Materials and methods

### Cell culture

U2OS and HEK293 cells were grown in Dulbecco’s Modified Eagle’s medium (DMEM, Sigma–Aldrich) supplemented with 10% fetal bovine serum (Sigma–Aldrich) and 100 I.U./ml penicillin–0.1 mg/ml streptomycin (Sigma–Aldrich) at 37°C in a humidified incubator with 5% CO_2_. Cells were tested monthly for Mycoplasma contamination. SPRTN was depleted from U2OS with siRNA (si3′UTR: 5′-GUCAGGAAGUUCUGGUUAA-3′, Mycrosynth) for 3 days before processing. Formaldehyde treatment in Hek293 cells was performed in complete DMEM medium for 30 min at 37°C.

### DPC isolation and detection

DPCs were isolated using a modified rapid approach [34, 41]. To detect H1.0 crosslinks, the equivalent of 15 μg of the isolated DNA was digested with benzonase (50 units, 1 h at 37°C) in a total of 400 μl. The entire volume was loaded onto a slot blot manifold (Bio-Rad) and applied to a nitrocellulose membrane. The membrane was processed for western blot against H1.0 (Proteintech, 17510-1-AP). Alternatively, 80 μg of DNA was digested with benzonase (50 units, 1 h at 37°C) in 1 ml; proteins were precipitated with trichloroacetic acid (TCA) and resolved by SDS–PAGE. After transfer to nitrocellulose and western blot, H1.0 DPCs were detected with the H1.0 antibody. For slot blot detection of dsDNA, 100–200 ng of DNA was incubated with 50 μg/ml proteinase K to digest the crosslinked proteins, diluted in Tris/Borate/EDTA (TBE) buffer and applied to a nylon membrane (GE Healthcare). After the western blot, DNA was detected with an anti-dsDNA antibody (Abcam, ab27156).

### Purification of recombinant proteins

Ubiquitin purification: To express and purify monoUb, the *Escherichia coli* BL21(DE3) strain carrying the pGEX-6P1-hUb plasmid was grown in LB medium at 37°C with constant shaking until OD_600_ reached ∼0.9, followed by isopropyl-β-d-thiogalactoside (IPTG) induction at a final concentration of 0.2 mM at 20°C overnight. The harvested cell pellet was resuspended in GST buffer (50 mM Tris–HCl, pH 7.4, 300 mM NaCl, 1 mM dithiothreitol (DTT)) containing 0.2 mM phenylmethanesulfonyl fluoride (PMSF) and one tablet EDTA-free protease inhibitor cocktail (ROCHE) and then processed by sonication. The lysate was centrifuged at 20 K rpm for 30 min. The above supernatant was loaded onto the pre-equilibrated self-packed column with 10 ml of Glutathione Sepharose High Performance (Cytiva). The protein was eluted with GST buffer containing 20 mM reduced GSH. The elution was added with HRV 3C protease for overnight cleavage at 4°C using a 3 ml of 3500 MWCO Slide-A-Lyzer Dialysis cassette (ThermoFisher) against 500 ml of GST buffer. The above overnight cleavage sample was loaded onto the GSH column to separate free GST from the tag-free monoUb. The flowthrough was collected and concentrated for SEC by HiLoad^™^ 16/600 Superdex^™^ 75 pg with the SEC buffer (50 mM HEPES, pH 7.4, 150 mM NaCl, 0.5 mM TCEP–HCl). The tag-free NEDD8 was expressed by pGEX2TK-NEDD8 following the same procedure above.

To express and purify His-tagged monoUb/M1-Ub_2_/M1-Ub_4_/M1-Ub_6_ (including variants), *E. coli* BL21(DE3) strain carrying the corresponding plasmid was grown in LB medium at 37°C with constant shaking until OD_600_ reached to ∼0.9. It was then induced by IPTG at a final concentration of 0.2 mM at 20°C overnight. The harvested cell pellet was resuspended in Equilibration Buffer (50 mM HEPES, pH 7.4, 300 mM NaCl, and 1 mM TCEP–HCl) with 0.2 mM PMSF and 30 mM imidazole and processed by sonication. The lysate was centrifuged at 20 K rpm for 30 min. The supernatant was loaded onto the pre-equilibrated 5 mL HisTrap FF column (Cytiva). The protein was eluted with Equilibration Buffer containing 300 mM imidazole. Elution was concentrated for the final SEC purification via the HiLoad^™^ 16/600 Superdex^™^ 75 pg column with the SEC Buffer (50 mM HEPES, pH 7.4, 150 mM NaCl, and 0.5 mM TCEP–HCl).

To express N^15^-labelled His-tagged monoUb, *E. coli* BL21(DE3) strain carrying the pNIC28-Ub plasmid was grown in N^15^-M9 medium (containing 6 g/l Na_2_HPO_4_, 3 g/l KH_2_PO_4_, 0.5 g/l NaCl, 2 g/l D-glucose, 0.7 g/l ^15^NH_4_Cl, 1× Gibco^®^ MEM vitamin solution, 0.1 M FeSO_4_, 1 M CaCl_2_, 1 M MgSO_4_, pH 7.4) at 37°C with constant shaking until OD_600_ reached to ∼1.0. It was then induced by IPTG at a final concentration of 0.2 mM at 20°C overnight. The purification procedure is the same as the unlabelled one from above, except that the SEC purification was performed with NMR buffer (22 mM phosphate, pH 7.0, 55 mM NaCl, and 1 mM DTT). N^15^-labelled M1-Ub_2_ was expressed and purified in a similar way, with the addition of HRV 3C protease for overnight cleavage to remove the His tag. The synthesis and purification of heterotopic/banched Ub chains ([Ub]_2_-^48,63^Ub-^63^Ub; [Ub]_2_-^48,63^Ub-^48^Ub) were performed as previously described [43].

SPRTN purification: Full-length SPRTN was expressed and purified following the previously published protocol [3]. To express GST-SPRTN-ΔC, *E. coli* Rosetta (DE3) strain carrying the pGEX-4T1-SPRTN-ΔC (Lys241AsnfsX8) plasmid was grown in LB medium (containing 100 μM ZnCl_2_) at 37°C with constant shaking until OD_600_ reached to ∼0.8. It was induced by IPTG at a final concentration of 0.2 mM at 18°C overnight. The harvested cell pellet was resuspended in ∼50 ml of GST High Salt Buffer (100 mM HEPES, pH 7.4, 500 mM NaCl, 10% glycerol, and 1 mM DTT) containing 0.2 mM PMSF, 0.1% DDM, 45 U/ml benzonase, and one tablet EDTA-free protease inhibitor cocktail (Roche) and then processed by sonication. The lysate was centrifuged at 20 K rpm for 30 min. The purification procedure is similar to that of GST-Ub, except that GST High Salt Buffer was used to equilibrate the column, and protein was eluted by GST High Salt Buffer containing 20 mM reduced GSH. The GST tag was not removed. The following SEC purification was performed by HiLoad^™^ 16/600 Superdex^™^ 75 pg with the GST SEC buffer (50 mM HEPES, pH 7.4, 500 mM NaCl, 10% glycerol, and 1 mM DTT). GST-MIU was purified similarly, except that the buffer used for SEC purification is 50 mM HEPES, pH 7.4, 150 mM NaCl, and 0.5 mM TCEP–HCl. SPRTN ZBD-BR was purified similarly with the addition of HRV 3C protease for overnight cleavage to remove the GST tag. Tag-free SPRTN ZBD-BR was then purified via the HiLoad^™^ 16/600 Superdex^™^ 75 pg column with SPRTN buffer (50 mM HEPES, pH 7.4, 500 mM NaCl, 1 mM MgCl_2_, 10% glycerol, and 1 mM TCEP–HCl).

To express the SPRTN core (including the L38A, L99A and E112Q variants), *E. coli* BL21(DE3) strain carrying pNIC28-SPRTN (26-240) plasmid (or variants) was grown in LB medium (containing 0.1 mM ZnCl_2_) at 37°C with constant shaking until OD_600_ reached to ∼1.0. It was then induced by IPTG at a final concentration of 0.5 mM at 18°C overnight. The harvested cell pellet was resuspended in SPRTN buffer containing 45 U/ml benzonase, 0.04 mg/ml Pefabloc, one tablet EDTA-free protease inhibitor cocktail (Roche), and processed by sonication. The lysate was centrifuged at 20 K rpm for 30 min. The supernatant was loaded onto the pre-equilibrated self-packed column with 20 ml of Ni-NTA resin (Invitrogen). The protein was eluted with SPRTN Buffer containing 300 mM imidazole. Elution was concentrated and added with TEV protease to remove the His tag. At the same time, it was dialysed overnight against 0.5 l SPRTN Buffer using an 8 kDa cut-off dialysis tube (Cytiva). The dialysed sample from TEV cleavage was loaded onto the pre-equilibrated 5 ml of HisTrap^™^ FF crude (Cytiva) by SPRTN Buffer. The flow-through was collected and further concentrated for the final purification by SEC. SEC was performed by loading the protein onto the HiLoad^™^ 16/600 Superdex^™^ 75 pg column with the SPRTN Buffer.

H1 purification: All the H1 proteins were expressed by the pCold cold shock system. *E. coli* Rosetta (DE3) strain carrying the corresponding plasmid was grown in LB medium at 37°C with constant shaking until OD_600_ reached ∼0.8. The culture was cold-shocked at 15°C for 30 min without shaking. It was induced by IPTG at a final concentration of 0.5 mM at 15°C for ∼24 h. The harvested cell pellet was resuspended in His-Eq Buffer (50 mM Tris–HCl, pH 7.4, 300 mM NaCl, 10% glycerol, and 0.5 mM TCEP–HCl) containing 0.2 mM PMSF, 30 mM imidazole, 1 tablet EDTA-free protease inhibitor cocktail (Roche), and then processed by sonication. The lysate was centrifuged at 20 K rpm for 30 min. The supernatant was loaded onto the pre-equilibrated 5 mL HisTrap FF column (Cytiva). H1 was eluted with His-Eq buffer containing 300 mM imidazole and further dialysed into His-IEX buffer (50 mM HEPES, pH 7.4, 150 mM NaCl, 10% glycerol, and 0.5 mM DTT). It was loaded onto the pre-equilibrated 5 mL HiTrap SP column (Cytiva). H1 was eluted using a linear 10 CV gradient of 100 mM to 1.5 M NaCl. The pure H1 fractions were pooled and dialysed into His-Eq buffer to reduce salt strength. Ub-H1, M1-Ub_4_-H1, and 2xISG15-H1 were purified in the same way with additional SEC purification by Superose^®^ 6 Increase 10/300 GL (Cytiva) with His-Eq Buffer.

PCNA (Proliferating Cell Nuclear Antigen) purification: To express and purify PCNA, *E. coli* BL21(DE3) strain carrying the pET16b-PCNA plasmid was grown in TB medium at 37°C with constant shaking until OD_600_ reached ∼0.7. It was then induced by IPTG at a final concentration of 0.5 mM at 16°C overnight. The harvested cell pellet was resuspended in 50 mM Tris–HCl pH 8.0, 100 mM NaCl, 10% glycerol, 1 mM EDTA, and 1 tablet EDTA-free protease inhibitor cocktail (Roche) and lysed by sonication. The lysate was centrifuged at 20 K rpm for 30 min. The supernatant was applied to a Q Sepharose Fast Flow column (GE Healthcare) pre-equilibrated in Buffer A (20 mM Tris–HCl pH 8.0, 10% glycerol). Protein was eluted using a linear 8 CV gradient of 100 mM to 1.5 M NaCl. Fractions containing PCNA were dialysed into Buffer B (20 mM Tris–HCl pH 8.0, 100 mM NaCl, and 10% glycerol) and loaded onto the pre-equilibrated HiPrep Heparin Fast Flow 16/10 (GE Healthcare). The column was washed with 2 CV Buffer B, followed by a 5 CV elution gradient from 0% to 100% of Buffer B containing 1.5 M NaCl. Fractions containing PCNA proteins were collected and concentrated for SEC by Superdex 200 Increase 10/300 GL (Cytiva) with GF buffer (20 mM Tris pH 8.0, 100 mM NaCl, 2.5% glycerol, and 1 mM TCEP-HCl).

### Cy5 labelling

To label naked H1 (Cys was introduced to either N- or C-terminal) with Cy5, ∼ 0.5 mg His-H1 was added with 10 μl of Cy5 (one vial of Cy5 was dissolved in 50 μl of dimethylsulfoxide (DMSO), Cytiva) in a 200 μl of total reaction with Labelling Buffer (50 mM HEPES, pH 7.4, 300 mM NaCl, and 0.5 mM TCEP–HCl). React at R.T. in the dark for ∼2.5h. The labelled protein was separated from the free dye by SEC using the Superose^®^ 6 Increase 10/300 GL (Cytiva) with the Labelling Buffer. Peak fractions were collected and concentrated/buffer-exchanged with new SEC buffer by a 10 K mini-concentrator (Millipore) to remove any residual dye. The reaction for labelling of the other Ub-fused H1 proteins was prepared the same as above, except that react in the dark at 4°C for ∼2 h. Due to the low stability of the Ub-fused H1 proteins, after incubation, the reaction was directly concentrated/buffer-exchanged with new SEC buffer several times by a 10 K mini-concentrator (Millipore) to remove any free dye.

To label SPRTN core (E112Q) with Cy5 for the MST measurements, 100 μl of SPRTN core (691.5 μM) was mixed with 10 μl of Cy5 (Cytiva, one vial was dissolved in 50 μl of DMSO) in 90 μl of SEC Buffer (50 mM HEPES, pH 7.4, 150 mM NaCl, and 0.5 mM TCEP–HCl). Incubate overnight in the dark at 4°C. The labelled protein was separated from the free dye by SEC using the Superose^®^ 6 Increase 10/300 GL (Cytiva) with the SEC Buffer. Peak fractions were collected and concentrated/buffer-exchanged with new SEC Buffer by a 10K mini-concentrator (Millipore) to remove any residual dye.

### Making dsDNAs

The ssDNAs (sequence provided in Key Resources in the Supplementary Data document; available at NAR online) were diluted in DEPC-treated water to a final concentration of 100 μM. To make dsDNA, an equal volume of ssDNA was mixed and incubated at 95°C for 10 min on a heating block. The mixture was then left on the heating block, switched off to allow cooling down with a gradient temperature drop until equilibrium with room temperature. dsDNA_dAT_15nt was made with 15nt-dA and 15nt-dT; dsDNA_20nt was made with Turner-20bp-F and Turner-20bp-R; dsDNA_23nt was made with WS-23bp-F and WS-23bp-R; dsDNA_100nt was made with OD4_Fw and OD4_Rev; dsDNA_20/23nt was made with Turner-20bp-F and WS-23bp-R; dsDNA_20nt_nicked was made with Turner-20bp-F, WS-10bp-R-nick1 and WS-10bp-R-nick2; dsDNA_23/10bp_fork was made with WS-23bp-fork-F and WS-23bp-fork-R.

### Making model H1-DPCs

The FAM-labelled dsDNA_20/23nt (FAM-Turner-20bp-F with Amide-WS-23bp-R, sequence provided in the Supplementary Data document; Key Resources Table, available at NAR online) was produced by annealing following the above protocol and then purified using a Zeba Spin desalting column (7 kDa, 3 ml, Thermo Scientific, 89889) pre-equilibrated with Crosslink Buffer (100 mM NaH_2_PO_4_, 150 mM NaCl, and 5 mM EDTA, pH 7.3) to remove any unbound ssDNA. The flow-through was collected and further incubated with SMCC at a final concentration of 5 mM at R.T. in the dark for 2 h. Any excess SMCC was removed by a new Zeba Spin desalting column. The flow-through containing dsDNA–SMCC was collected for conjugating with proteins (H1, Ub_4_-H1). Five-fold excess of dsDNA–SMCC was mixed with the protein and incubated overnight at 4°C in the dark. The mixture was applied to IEX by a 1 ml of Resource S column (Cytiva) pre-equilibrated with IEX buffer A (50 mM HEPES pH 7.4, 150 mM NaCl, 10% glycerol, and 0.5 mM DTT). The protein conjugated to dsDNA was eluted by IEX buffer B (50 mM HEPES pH 7.4, 1 M NaCl, 10% glycerol, and 0.5 mM DTT) and concentrated by a 3K mini-concentrator (Millipore) as the final DPC product.

### Making monoUb-PCNA

Monoubiquitination of PCNA *in vitro* was adopted from a previously published protocol [42], with below optimization. Ube1 (1 μM), Ubc5Hc-S22R (10 μM), His-monoUb (30 μM), PCNA (2 μM), and ATP (10 mM) were prepared in MMT working buffer (50 mM MMT, pH 9, 25 mM NaCl, 3 mM MgCl_2_, and 0.5 mM TCEP–HCl) and incubated overnight at 30°C. The sample was centrifuged, where the supernatant was loaded onto the Superose^®^ 6 Increase 10/300 GL (Cytiva) with SEC Buffer (50 mM HEPES, pH 7.4, 150 mM NaCl, and 0.5 mM TCEP–HCl) to separate monoUb-PCNA from other ubiquitination components.

### SPRTN cleavage assay

For the SPRTN cleavage towards free H1 substrates, recombinant SPRTN (or variants) (2 μM) were incubated with H1 (1 μM) in the absence or presence of dsDNA_20/23nt (2.7 μM) in combination with Ubs or Ubs (2 μM, unless specified otherwise) with the indicated time at 30°C in the Assay Buffer (25 mM Tris–HCl, pH 7.4, 150 mM NaCl, and 0.5 mM TCEP–HCl). For the SPRTN cleavage towards H1-DPC substrates, recombinant SPRTN (or SPRTN core) (2 μM) was incubated with H1-DPC substrates (∼0.1 μM) in combination with M1-Ub_4_ (2 μM) with the indicated time at 30°C in the assay buffer. Details can be found in each corresponding figure legend.

### Microscale thermophoresis

A serial dilution of monoUb (from 4754.8 to 0.15 μM) and 1.2 μM Cy5-labelled SPRTN core was prepared in a total volume of 20.5 μl with microscale thermophoresis (MST) buffer (50 mM HEPES, pH 7.4, 150 mM NaCl, and 0.5 mM TCEP–HCl). Each sample was loaded into the capillary (Nanotemper) for measurement with 20% MST power on an MST instrument (Monolith NT.115, Nanotemper). The data were analysed and plotted by the NT Analysis Software (1.5.41, Nanotemper). All the analyses were done using the “Thermophoresis + Temperature Jump” method. The fluorescent value is fixed to 1.2 μM for the fitting.

### Isothermal titration calorimetry

Protein samples were dialysed against ITC Buffer (50 mM HEPES, pH 7.4, 50 mM NaCl, and 0.5 mM TCEP–HCl) overnight at 4°C using the Mini Dialysis Kit (1 KDa cut-off, Cytiva) before measurements. All the ITC measurements were conducted in a MicroCal PEAQ-ITC calorimeter (Malvern) with a standard 13-injection titration program (1st injection: 0.4 μl/injection, duration: 0.8 s, spacing time: 150 s; the rest of the injections: 3 μl/injection, duration: 6 s, spacing time: 150 s) at 25°C, with a reference power at 10 μCal/s. Stir speed is 750 rpm. Initial delay is 60 s. The data are processed and analysed using the MicroCal PEAQ-ITC Analysis Software with a one-site binding model.

### Surface plasmon resonance

Surface plasmon resonance (SPR) measurements were performed on a Biacore^™^ S200 (GE Healthcare). SPRTN PIP-UBZ was immobilized onto a Series S sensor chip CM5 (Cytiva) by an amine coupling kit (Cytiva) at pH 5.0 until the response on the surface reached ∼1000 RU. A serial dilution of the analytes (monoUb, PCNA and monoUb-PCNA) was prepared on a 96-well plate. Analytes were injected onto the chip at a flow rate of 35 μl/min with 60 s association and 200 s dissociation. Running Buffer used for SPR contains 50 mM HEPES, pH 7.4, 50 mM NaCl, and 0.5 mM TCEP–HCl. The signal from the reference channel was subtracted from the signal obtained from the sample channel. The data were fitted by the Biacore S200 Evaluation software (GE Healthcare) using a steady-state affinity binding model with report points taken at 8 s after injection start.

### NMR spectroscopy

^15^N-labelled proteins (His-monoUb, M1-Ub_2_) were prepared at a concentration of 100 μM in NMR buffer (22 mM phosphate, pH 7.0, 55 mM NaCl, and 1 mM DTT) containing 5% D_2_O and 0.05% NaZ. NMR spectra were recorded on a 950 MHz spectrometer (Bruker Avance III HD console) equipped with a high-sensitivity 5 mm TCl cryoprobe at 25°C.

For the titration between ^15^N-labelled His-monoUb (or ^15^N-labelled M1-Ub_2_) and SPRTN core, a series of titrations is made by the addition of unlabelled SPRTN core to the ^15^N-labelled His-monoUb (or ^15^N-labelled M1-Ub_2_). HSQC spectra were recorded. Spectra were initially processed using TopSpin 3.6 and then analysed and plotted using MestReNova. The volume of the peaks from monoUb or M1-Ub_2_ that are completely broadened upon the addition of SPRTN core or UBZ is defined as 0.

### Size-exclusion chromatography coupled with multiple-angle laser light scattering

UBZ or UBZ* were prepared at 3.0 mg/ml, and then 100 μl of each protein was separately applied to the pe-equilibrated SEC column (Superdex^®^ 75 10/300 Increase, Cytiva) using the buffer containing 50 mM HEPES pH 7.4, 150 mM NaCl, and 0.5 mM TCEP–HCl. The protein eluted from SEC was monitored using a DAWN HELEOS-II 18-angle light scattering detector (Wyatt Technologies), a U9-M UV/Vis detector (Cytiva), and an Optilab T-rEX refractive index monitor (Wyatt Technologies). Data were analysed by using Astra v7 (Wyatt Technologies) with a refractive increment value of 0.185 ml/g.

### Data analysis

Multiple sequence alignment was performed by CLUSTAL (1.2.4).

For the kinetic data from the SPRTN cleavage assay, the signal from each time point was normalized to the “0 min” time point within each condition. Kinetic data were fitted with simple linear regression or one-phase exponential decay-least squares fit (Prism). Significant analysis from SPRTN cleavage assay was performed using unpaired *t*-test or ANOVA (Prism). Details can be found in each corresponding figure legend.

## Results

### SPRTN proteolysis towards the substrate is activated by single-stranded (ss) and double-stranded (ds) DNA

To gain mechanistic insights into SPRTN-dependent substrate proteolysis, we chose histone H1 as a model substrate. Histone H1 has been widely used to study SPRTN proteolysis *in vitro* [3, 31]. To confirm that histone H1 is a relevant substrate for SPRTN *in vivo*, we depleted SPRTN in U2OS cells (Supplementary Fig. S1A). SPRTN depletion by siRNA caused increased covalent attachment of histone H1 to genomic DNA when analysed by the RADAR assay, a well-recognized method to measure DPCs [41, 43] (Supplementary Fig. S1B). We also found that histone H1 is an abundantly crosslinked protein in response to formaldehyde (Supplementary Fig. S1C). Interestingly, formaldehyde treatment further induced the accumulation of high molecular weight species, which react with an anti-H1.0 specific antibody (Supplementary Fig. S1C), most likely post-translationally modified histone H1 [34, 38].

**Figure 1.**
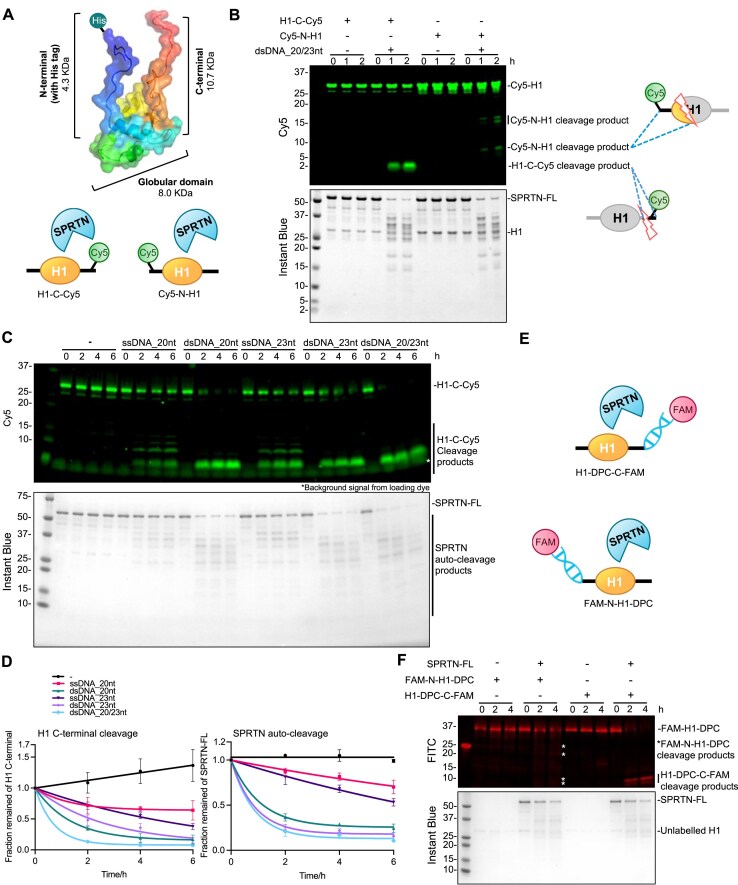
SPRTN proteolysis is activated by both ssDNA and dsDNA. (**A**) Design of the Cy5-labelled H1 substrates (Cy5-C-H1 and Cy5-N-H1) for SPRTN cleavage assay. The size of the H1 N-terminal (including the His tag), C-terminal and globular domain is indicated, respectively. (**B**) SPRTN cleavage assay towards the model H1 substrates. Recombinant full-length SPRTN (2 μM) and Cy5-labelled H1 substrates (H1-C-Cy5 or Cy5-N-H1, 1 μM) were incubated in the absence or presence of dsDNA_20/23nt (2.7 μM) with the indicated time at 30°C. The reaction was analysed by SDS–PAGE followed by Cy5-scanning on Typhoon FLA 9500 (GE Healthcare) and Instant Blue staining. Representative figure from three repeats. (**C**) SPRTN kinetics towards H1-C-Cy5. Recombinant full-length SPRTN (2 μM) and H1-C-Cy5 (1 μM) were incubated with indicated ssDNAs or dsDNAs (2.7 μM) at the indicated time at 30°C. The reaction was analysed by SDS–PAGE followed by Cy5-scanning on an iBright 1500 imaging system (Invitrogen) and Instant Blue staining. Representative figure from three repeats. (**D**) Cleavage kinetics of the full-length H1-C-Cy5 substrate (C-terminal cleavage rate) and the full-length SPRTN (auto-cleavage rate) from Fig. 1C. Cy5 and SPRTN-FL signals were analysed by the iBright Analysis Software (Invitrogen). Kinetic data were fitted with one phase exponential decay-least squares fit (Prism); *n* = 3; error bar, SD. (**E**) Design of the FAM-dsDNA labelled H1-DPC substrates (H1-DPC-C-FAM and FAM-N-H1-DPC) for SPRTN cleavage assay. (**F**) SPRTN cleavage assay towards the model H1-DPC substrates. Recombinant full-length SPRTN (2 μM) and the model H1-DPC substrates (H1-DPC-C-FAM or FAM-N-H1-DPC, ∼0.1 μM) were incubated with the indicated time at 30°C. The reaction was analysed by SDS–PAGE followed by FITC-scanning on an iBright 1500 imaging system (Invitrogen) and Instant Blue staining. Representative figure from two repeats. See also Supplementary Fig. S1.

To monitor SPRTN’s proteolytic activity on H1 and bypass the limitations of western blot detection of its cleavage products [3, 22, 31], we developed a fluorescence-based *in vitro* assay (Fig. 1A). N- or C- terminally Cy5-labelled histone H1 (Cy5-N-H1 and H1-C-Cy5 hereafter) were generated to monitor SPRTN’s enzymatic activity (Fig. 1A, lower panel).

As SPRTN’s activity is strictly DNA-dependent, we first used dsDNA with a 3-nt overhang (dsDNA 20/23 nt) as it was reported to be the best DNA structure to activate SPRTN proteolysis [3]. It was designed based on known ssDNA sequences that do not precipitate histone H1 when used in the SPRTN proteolysis assay [44]. Importantly, our Cy5-fluorescence-based assays provided a higher resolution of histone H1 cleavage products by SPRTN proteolysis than previously reported [3, 22, 31] (Fig. 1B). SPRTN was shown to cleave mostly unstructured regions of the core histones, namely the histone tails [19]. Likewise, SPRTN preferably cleaved the more disordered C-terminus (Fig. 1B). The cleavage at the N-terminus of histone H1 was rate-limited. However, multiple cleavage products were observed, which indicate that SPRTN cleaves not only the unstructured N-terminal tail (N-terminal tail is 4.3 kDa including the His tag, Fig. 1A) but also structured globular regions of H1 (Fig. 1B, the last two lanes), as one of the cleavages products are between 5 and 10 kDa.

There is an ongoing debate regarding how different DNA templates activate SPRTN. A dogma has been accepted that ssDNA activates SPRTN towards the substrate and blunt-ended dsDNA towards auto-cleavage, consequently causing SPRTN inactivation. However, it has been shown that SPRTN cleaves core histones, denatured TOP1/TOP2 and CHK1 in the presence of 100-nt dsDNA with blunt ends [19, 25]. In contrast, other studies demonstrated that SPRTN proteolysis towards histone H1 cannot be activated by blunt-ended or circular dsDNAs (ranging from 60 nt to 5.4 kb) [3, 31], and that the presence of either ssDNA or dsDNA determines better substrate cleavage or auto-cleavage, respectively [22]. To clear up this controversy, we took advantage of our assay, where cleaving the C-terminal fluorescently labelled H1 (H1-C-Cy5, Fig. 1C) provides a clear and robust readout of SPRTN activity. Different ssDNAs and dsDNAs were screened based on our fluorescence assay, and both the cleavage of histone H1 and the auto-cleavage of SPRTN were monitored (Fig. 1C and D). Results demonstrated that blunt-ended dsDNAs (without overhang) could also activate SPRTN proteolysis, as a very small Cy5-visible cleavage product around 2 kDa appeared, which corresponds to the very end of H1 C-terminal (“dsDNA_20nt”, “dsDNA_23nt”, Fig. 1C and Supplementary Fig. S1D). In addition, ssDNA facilitated SPRTN attack into the structured region of histone H1 (“ssDNA_20nt”, ssDNA_23nt”, Fig. 1C and Supplementary Fig. S1D) as additional cleavage fragments with a size roughly above 5 kDa appeared (Supplementary Fig. S1D). In line with a previous study [3], dsDNA, but not ssDNA, induced strong SPRTN auto-cleavage within 2 h (Fig. 1C and Supplementary Fig. S1D). Still, ssDNA governs the specificity towards substrates over self-cleavage.

Notably, the SPRTN proteolysis kinetics tested by various DNA structures (ss, ds, overhangs) showed the most rapid cleavage towards the C-terminal tail of histone H1 was achieved by the ss/dsDNA overhang (“dsDNA_20/23nt”, Fig. 1C and D, and Supplementary Fig. S1D), which is in agreement with previous reports [3]. Longer circular ss- and ds-DNAs were also tested. Intriguingly, circular dsDNA could not activate SPRTN under 150 mM NaCl, which is close to the physiological condition, whereas the activation from circular ssDNA is independent of salt concentration (Supplementary Fig. S1F). Both are in line with previously published data [3, 22]. SPRTN activation is still in favour of ss/dsDNA junctions. However, although the ss/dsDNA junction structure (20/23 nt) was the best activator of SPRTN proteolysis towards both substrate-cleavage and auto-cleavage, the cleavage kinetics was still relatively slow (∼2 h), indicating SPRTN is not a very efficient enzyme (Fig. 1C and D).

A model H1-DPC substrate was also developed where the FAM-labelled dsDNA was covalently attached to either the N- or C-terminal of H1 (Fig. 1E). Again, SPRTN proteolysis towards the model H1-DPCs indicated a cleavage pattern similar to free H1 (please compare Fig. 1F with Fig. 1B).

Altogether, we established a sensitive fluorescence-based assay to monitor SPRTN proteolysis on a genuine DPC substrate, histone H1, *in vitro*. We demonstrated that ss and dsDNA structures activate SPRTN proteolytic activity towards itself and the substrate. In addition, we confirmed that the 3-nt overhang dsDNA structure (20/23 nt) was the best activator of SPRTN proteolysis. SPRTN cleaves its substrate and itself on this DNA structure with similar efficiency (Fig. 1C and D, last four lanes), suggesting its inactivation during substrate processing and no substrate specificity.

### Ubiquitin chains rapidly activate SPRTN proteolysis

**Figure 2.**
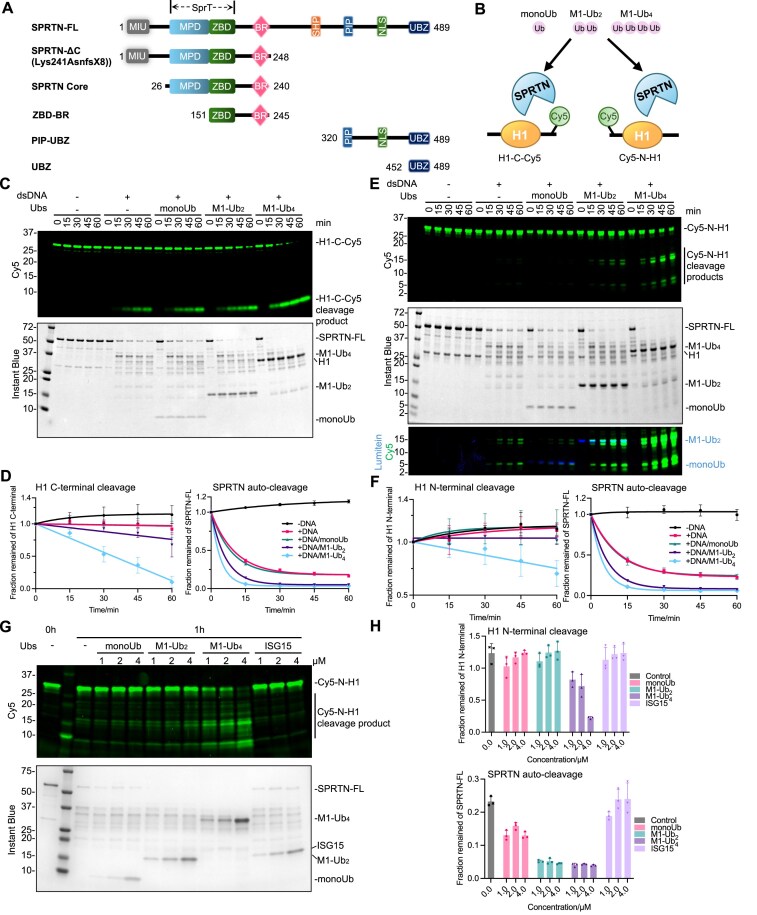
Ubiquitin chains rapidly activate SPRTN proteolysis. (**A**) Schematics of SPRTN domain structure. MIU, Motif Interacting with Ub-binding domain; SprT, the metalloprotease domain similar to that of the *E. coli* SprT protein; MPD: Metalloprotease domain; ZBD: Zn-binding domain; BR, basic region; SHP, p97 or VCP-binding motif; PIP, PCNA interaction peptide; NLS: nuclear localization signal; UBZ, ubiquitin-binding zinc finger. (**B**) Schematics of Ub titration to SPRTN cleavage assay with Cy5-labelled H1 substrates (Cy5-C-H1 or Cy5-N-H1). (**C**) SPRTN cleavage assay towards H1-C-Cy5 in the presence of Ubs. Recombinant full-length SPRTN (2 μM) was incubated with H1-C-Cy5 (1 μM) in the absence or presence of dsDNA_20/23nt (2.7 μM) in combination with Ubs (monoUb, M1-Ub_2_, M1-Ub_4_, all at 2 μM) with the indicated time at 30°C. Representative figure from three repeats. (**D**) Cleavage kinetics of the full-length H1-C-Cy5 substrate (C-terminal cleavage rate) and the full-length SPRTN (auto-cleavage rate) from Fig. 2C; *n* = 3; error bar, SD. (**E**) SPRTN cleavage assay towards Cy5-N-H1 in the presence of Ubs. Recombinant full-length SPRTN (2 μM) was incubated with Cy5-N-H1 (1 μM) in the absence or presence of dsDNA_20/23nt (2.7 μM) in combination with Ubs (monoUb, M1-Ub_2_, M1-Ub_4_, all at 2 μM) with the indicated time at 30°C. The overlay of Cy5 and Lumitein signals (both with stronger contrast) is also displayed to show the similar size of the H1 cleavage product and monoUb. Representative figure from four repeats. (**F**) Cleavage kinetics of the full-length Cy5-N-H1 substrate (N-terminal cleavage rate) and the full-length SPRTN (auto-cleavage rate) from Fig. 2E. *n* = 4; error bar, SD. (**G**) SPRTN cleavage assay towards Cy5-N-H1 with Ubs or ISG15. Recombinant full-length SPRTN (2 μM) and Cy5-N-H1 (1 μM) were incubated with indicated Ubs (monoUb, M1-Ub_2_, M1-Ub_4_) or ISG15 at various concentrations (1–4 μM) in the presence of dsDNA_20/23nt (2.7 μM) for 1 h at 30°C. The reactions were analysed with SDS–PAGE followed by Cy5-scanning on an iBright 1500 imaging system (Invitrogen) and Instant Blue staining. Representative figure from three repeats. (**H**) Quantification of the signal from the full-length Cy5-N-H1 substrate (N-terminal cleavage rate) and the full-length SPRTN (auto-cleavage rate) from Fig. 2G; *n* = 3; error bar, SD. The reactions from Fig. 2C and E were analysed with SDS–PAGE followed by Cy5-scanning on Typhoon FLA 9500 (GE Healthcare) and Instant Blue staining. Cy5 signals from Fig. 2C and E were analysed by ImageJ. Cy5 signals from Fig. 2G were analysed by the iBright Analysis Software (Invitrogen). SPRTN-FL signals from Fig. 2C, E, and G were analysed by the iBright Analysis Software (Invitrogen). Kinetic data from Fig. 2D and F were fitted with one phase exponential decay-least squares fit (Prism), respectively. See also Supplementary Fig. S2.

The slow kinetics of substrate cleavage (Fig. 1 and Supplementary Fig. S1) suggests that SPRTN is a very inefficient enzyme. SPRTN is also a promiscuous enzyme that cleaves many DNA-bound proteins [3, 19, 22, 25, 31]. This is counterintuitive, considering its role as a DNA replication-coupled enzyme for the timely resolution of DPCs in front of the DNA replication fork [19, 21, 25]. The replication fork is enriched with the replisome, whose unspecific cleavage would harm the proliferative cell [45]. All these facts suggest that additional factors must regulate SPRTN enzymatic activity and specificity. As SPRTN possesses the Ub-binding domain Zn finger (UBZ) at its C-terminal (Fig. 2A) and DPCs are heavily ubiquitinated [27, 34], we hypothesized that ubiquitin or ubiquitination on its substrates could play a regulatory role for SPRTN proteolysis and specificity.

Using the above-established H1-C-Cy5 as a substrate for SPRTN proteolysis in the presence of 3-nt overhang dsDNA (20/23 nt), we added either monoUb, M1-linked diUb (M1-Ub_2_) or M1-linked tetraUb (M1-Ub_4_) to test our hypothesis (Fig. 2B). Interestingly, the addition of M1-Ub_2_, but not monoUb, in the reaction weakly increased the cleavage capacity of SPRTN on histone H1 (Fig. 2C and D), whereas the addition of M1-Ub_4_ in the reaction induced strong activation of SPRTN proteolytic activity towards itself and the substrate histone H1. Almost half of the C-terminal tail from H1 was cleaved in 30 min and almost 100% in 60 min (Fig. 2C and D). As ubiquitin itself could not activate SPRTN without DNA in the reaction (Supplementary Fig. S2A), this indicates that ubiquitin activation is the second tier in regulating SPRTN proteolysis. At the same time, DNA is still a prerequisite for SPRTN activation.

A similar Ub-chain activation effect was observed when Cy5-N-H1 was used as the substrate (Fig. 2E and F). M1-Ub_4_ significantly enhanced SPRTN proteolysis into the structured histone H1 globular domain, as visible by the decreased full-length H1 and the increased levels of multiple lower molecular weight cleavage products (Fig. 2E, the last four lanes). Ubiquitin chain length was essential to sufficiently activate SPRTN, as monoUb or M1-Ub_2_ at higher concentrations could not efficiently activate SPRTN towards Cy5-N-H1 compared to M1-Ub_4_ at a lower concentration (Fig. 2G and H).

Notably, the ubiquitin-activation of SPRTN protease was not linkage-specific as all eight diUb chains, including the M1, K6, K11, K27, K29, K33, K48, and K63 linkages, could enhance SPRTN activation, among which M1-Ub_2_ displays the best activation on SPRTN proteolysis (Supplementary Fig. S2B and C). Furthermore, all tested tetraUb chains rapidly promoted C-terminal Histone H1 cleavage, among which K6- and K63-Ub_4_ have relatively faster kinetics compared to other tetraUb chains (Supplementary Fig. S2D). Two branched tetraUb chains were also tested [46], showing a similar activation effect to that of unbranched chains, indicating that SPRTN is not selective towards the Ub chain types (homotypic and heterotypic/branched Ub chains) (Supplementary Fig. S2E).

It’s also worth noting that this activation effect was Ub-specific, as the addition of the Ub-like protein ISG15 in the reaction, which resembles the structure of the linear diUb molecule, did not activate SPRTN (Fig. 2G and H, and Supplementary Fig. S2B, C, F, and G). The addition of other Ub-like proteins, such as SUMO1, SUMO2, SUMO3, SUMO2 chains (2–7), or NEDD8 to the reaction did not stimulate SPRTN proteolytic activity (Supplementary Fig. S2F and G). Altogether, these results showed that Ub chains, regardless of their linkage type, activate SPRTN protease activity. This effect is more pronounced if the Ub chains are longer (see Fig. 4E and F).

### The N-terminal core region of SPRTN is sufficient for its ubiquitin-dependent activation

**Figure 3.**
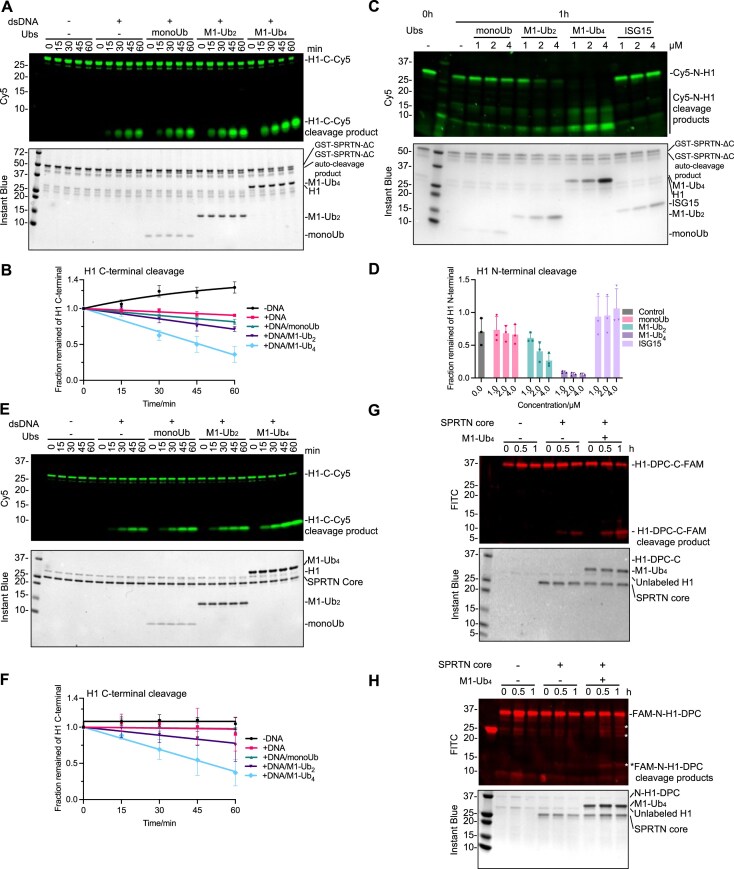
SPRTN core region is sufficient for the rapid activation of SPRTN protease. (**A**) SPRTN-ΔC cleavage assay towards H1-C-Cy5 in the presence of Ubs. Recombinant GST-SPRTN-ΔC (2 μM) and H1-C-Cy5 (1 μM) were incubated in the absence or presence of dsDNA_20/23nt (2.7 μM) in combination with Ubs (monoUb, M1-Ub_2_, M1-Ub_4_, all at 2 μM) with the indicated time at 30°C. Representative figure from three repeats. (**B**) Cleavage kinetics of the full-length H1-C-Cy5 substrate (C-terminal cleavage rate) from Fig. 3A; *n* = 3; error bar, SD. (**C**) SPRTN-ΔC cleavage assay towards Cy5-N-H1 in the presence of Ubs or ISG15. Recombinant GST-SPRTN-ΔC (2 μM) and Cy5-N-H1 (1 μM) were incubated with indicated Ubs (monoUb, M1-Ub_2_, M1-Ub_4_) or ISG15 at various concentration (1–4 μM) in the presence of dsDNA_20/23nt (2.7 μM) for 1 h at 30°C. Representative figure from three repeats. (**D**) Quantification of the signal from the full-length Cy5-N-H1 substrate (N-terminal cleavage rate) from Fig. 3C; *n* = 3; error bar, SD. (**E**) SPRTN core cleavage assay towards H1-C-Cy5 in the presence of Ubs. Recombinant SPRTN core (2 μM) and H1-C-Cy5 (1 μM) were incubated in the absence or presence of dsDNA_20/23nt (2.7 μM) in combination with Ubs (monoUb, M1-Ub_2_, M1-Ub_4_, all at 2 μM) with the indicated time at 30°C. Representative figure from three repeats. (**F**) Cleavage kinetics of the full-length H1-C-Cy5 substrate (C-terminal cleavage rate) from Fig. 3E; *n* = 3; error bar, SD. (**G**and **H**) SPRTN core cleavage assay towards the model H1-DPC substrates with M1-Ub_4_. Recombinant SPRTN core (2 μM) and the model H1-DPC substrates H1-DPC-C-FAM (G) or FAM-N-H1-DPC (H) (∼0.1 μM) were incubated in the absence or presence of M1-Ub_4_ (2 μM) with the indicated time at 30°C. The reaction was analysed by SDS–PAGE followed by FITC-scanning on an iBright 1500 imaging system (Invitrogen) and Instant Blue staining. Representative figure from two repeats. The reactions from Fig. 3A and E were analysed by SDS–PAGE, followed by Cy5 Scanning on Typhoon FLA 9500 (GE Healthcare) and Instant Blue staining. Cy5 signals were analysed by ImageJ. The reactions from Fig. 3C were analysed by SDS–PAGE followed by Cy5-scanning on an iBright 1500 imaging system (Invitrogen) and Instant Blue staining. Cy5 signals were analysed by the iBright Analysis Software (Invitrogen). Kinetic data from Fig. 3B and F were fitted with a one-phase exponential decay-least squares fit (Prism), respectively. See also Supplementary Fig. S3.

SPRTN contains a C-terminal UBZ domain (Fig. 2A), essential for SPRTN recruitment to DPC substrates and colocalization at the stalled DNA replication fork [34, 47–49]. To investigate if the UBZ domain is involved in ubiquitin-dependent SPRTN activation, we purified a disease-associated variant of SPRTN (SPRTN-ΔC; 1–248 aa) where the C-terminal, including the UBZ domain, was deleted (Fig. 2A). Although SPRTN-ΔC has slightly reduced intrinsic activity [19, 22], for yet unknown reason, surprisingly, SPRTN-ΔC can still be activated by ubiquitin chains (Fig. 3A and B) in a dose-dependent manner (Fig. 3C and D). This set of experiments demonstrates that the UBZ domain is not required for the Ub-mediated activation of SPRTN proteolytic activity.

Bioinformatic analysis of the SPRTN protein predicted an additional Motif Interacting with Ubiquitin (MIU) domain located at the first helix of SPRTN (1–20 aa) [11] (Fig. 2A). However, the ubiquitin-binding ability of this putative MIU domain was never experimentally proven. To test the role of the MIU domain in the Ub-dependent activation, we purified a SPRTN variant lacking both the MIU and UBZ domains (ΔMIU, ΔC: 26–240 aa, named SPRTN core region hereafter) (Fig. 2A). Unexpectedly, the SPRTN core region was still activated by M1-Ub_4_ with kinetics that mimicked SPRTN-ΔC (roughly 60% cleavage within 60 min) (Fig. 3E and F). The titration of the SPRTN core region, histone H1 and M1-Ub_4_, in the molar ratio of 2:1:2 caused the most robust activation of the protease, detectable already after only 15 min (Supplementary Fig. S3A and B). We also confirmed the ubiquitin-dependent activity of the SPRTN core region towards the model DPC substrates where H1 was covalently attached to FAM-labelled dsDNA in the presence of M1-Ub_4_ (Fig. 3G and H).

In line with the MIU domain being dispensable in the *in vitro* substrate cleavage assays, ITC demonstrated that the putative MIU alone could not interact with monoUb or Ub chains (Supplementary Fig. S3C). Competition between the MIU peptide (or GST-MIU) and M1-Ub_4_ in the SPRTN auto-cleavage assay was also not observed (Supplementary Fig. S3D and E), indicating that SPRTN MIU does not bind Ub and is not responsible for Ub-dependent SPRTN activation.

Altogether, this suggests that the SPRTN core region alone, which possesses an intrinsic protease domain (MPD), is directly activated by Ub chains, and neither the UBZ nor MIU domain is involved in Ub-dependent SPRTN protease activation.

### The N-terminal SPRTN core region interacts with Ub over a classical Ub Ile44 hydrophobic patch

**Figure 4.**
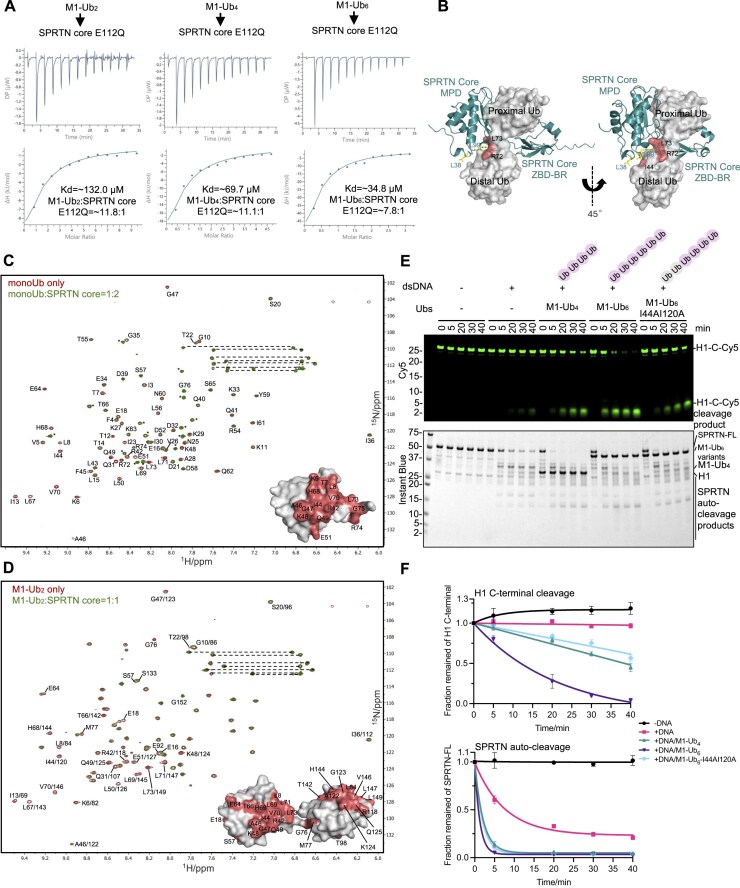
SPRTN core interacts with I44 patch of Ub in an avidity manner. (**A**) ITC analysis of the binding between SPRTN core (E112Q) and Ubs (M1-Ub_2_, M1-Ub_4_, M1-Ub_6_). The dissociation constant (*K*_d_) and stoichiometry of binding (N) are indicated here and summarized in Supplementary Table S1. (**B**) ColabFold prediction of the interaction between SPRTN core and M1-Ub_2_. (**C**) Superimposition of the 950 MHz ^1^H-^15^N HSQC spectra of monoUb alone (in red contours) or with the addition of SPRTN core at a molar ratio of 1:2 (in green contours). Spectra were plotted using the MestReNova software. Residues with complete broadening are highlighted in salmon on the monoUb surface (PDB: 1UBQ). The signals were assigned according to previously reported chemical shifts of ubiquitin (BMRB entry: 17769). *Signals from the N-terminal His tag on monoUb. (**D**) Superimposition of the 950 MHz ^1^H-^15^N HSQC spectra of M1-Ub_2_ alone (in red contours) or with the addition of SPRTN core at a molar ratio of 1:1 (in green contours). Spectra were plotted by using MestReNova software. Residues with complete broadening are highlighted in salmon on the M1-Ub_2_ surface (PDB: 2W9N). The signals were assigned according to previously reported chemical shifts of M1-Ub_2_ (BMRB entry: 26709). (**E**) Validation of M1-Ub_6_ I44 mutant on the effect of SPRTN activation. Recombinant full-length SPRTN (2 μM) and H1-C-Cy5 (1 μM) were incubated in the absence or presence of dsDNA_20/23nt (2.7 μM) in combination with Ubs (M1-Ub_4_, M1-Ub_6_, M1-Ub_6_-I44AI120A, all at 2 μM) with the indicated time at 30°C. The reactions were analysed with SDS–PAGE followed by Cy5-scanning on Typhoon FLA 9500 (GE Healthcare) and Instant Blue staining. Representative figure from three repeats. (**F**) Kinetics of the full-length H1-C-Cy5 substrate (C-terminal cleavage rate) and the full-length SPRTN (auto-cleavage rate) from Fig. 4E. Cy5 signals were analysed by ImageJ. SPRTN-FL signals were analysed by the iBright Analysis Software (Invitrogen). Kinetic data were fitted with one phase exponential decay-least squares fit (Prism); *n* = 3; error bar, SD. See also Supplementary Fig. S4.

As the N-terminally located MIU domain neither binds Ub nor is involved in SPRTN activation, we focused on the SPRTN core region (26–240 aa) (Fig. 2A). The SPRTN core region is composed of the metalloprotease domain (MPD), where the catalytic active centre is located, the ZBD, and the basic DNA-binding region (BR) domain, which is essential for SPRTN protease activation by binding to DNA [31] (Fig. 2A). To find out how ubiquitin chains activate SPRTN protease activity, we measured affinity, stoichiometry, and enthalpy changes by ITC between the SPRTN core region and monoUb, M1-Ub_2_, M1-Ub_4_ or M1-Ub_6_ chains (Fig. 4A, and Supplementary Fig. S4A and Supplementary Table S1). Enzymatically inactive SPRTN core region (E112Q) for ITC was used to avoid any possible auto-cleavage in the reaction. While binding between the SPRTN core region and monoUb was undetectable by ITC; however, the affinity of the SPRTN core region for M1-Ub_2_ was observed at ∼132 μM, and it further increased to ∼70 μM for M1-Ub_4_ and ∼35 μM for M1-Ub_6_ (Fig. 4A, and Supplementary Fig. S4A and Supplementary Table S1). These data strongly suggests that SPRTN increases its affinity for longer ubiquitin chains through avidity effects. As the Ub chains get longer, the SPRTN core has more opportunities to re-associate with the neighbouring ubiquitin moieties from the same chain when it becomes disassociated, thereby enhancing the affinity.

To narrow down which part of the SPRTN core region binds Ub chains, we purified a SPRTN truncated variant containing only the ZBD-BR domains (Fig. 2A). ITC indicated that the ZBD-BR domain did not interact with monoUb, M1-Ub_2_ or M1-Ub_4_ chains (Supplementary Fig. S4A), suggesting that the MPD domain is essential for the Ub-chain binding and SPRTN protease activation. We then used AlphaFold2 to predict the interaction between the SPRTN core region and M1-Ub_2_ (Fig. 4B and Supplementary Fig. S4B). AlphaFold2 showed that the SPRTN core region binds M1-Ub_2_ with high confidence (Supplementary Fig. S4B) and predicted a binding interface at the MPD domain that engages with the ubiquitin isoleucine 44 (I44) hydrophobic patch, a crucial surface for the interaction with Ub-binding domains (Fig. 4B).

The canonical binding mode of ubiquitin via the I44 patch from the above prediction suggests that there might be a very weak binding between monoUb and the SPRTN core region. Regardless of the undetectable affinity between the SPRTN core and monoUb by ITC (Supplementary Fig. S4A), the analysis by MST, a more sensitive biophysical method for assessing protein–protein interactions, suggested that the interaction between SPRTN core and monoUb indeed exists, but with an extremely weak affinity at ≥769 μM (Supplementary Fig. S4C).

We switched to NMR spectroscopy to further explore the physical interaction between the SPRTN core and monoUb, which can extract more detailed information on the binding interfaces, intermolecular affinity, and binding-induced conformational changes for complexes formed in solution. Encouragingly, NMR spectroscopy detected this weak protein–protein interaction in a higher resolution at the single-residue level. The overlaid spectra for the monoUb bound/unbound with SPRTN core confirmed a conserved perturbed surface centred on the I44 patch from ubiquitin (Fig. 4C). A similar perturbed pattern from M1-Ub_2_ upon the SPRTN core titration was also illustrated, suggesting the same binding mode between Ub and the SPRTN core region (Fig. 4D).

To validate the relevance of the identified interaction between Ub and SPRTN, we mutated I44 to alanine in either the distal Ub (I44A), the proximal Ub (at a position of aa 120; I120A) or on both Ub moieties (I44A; I120A) of M1-Ub_2_ and monitored how these mutations affect SPRTN’s affinity to ubiquitin and its proteolytic activity. Specifically, ITC showed that the interaction between the SPRTN core region and the M1-Ub_2_ I44A variants was undetectable, indicating that the affinity is reduced to at least the level of monoUb (Supplementary Fig. S4D). From the Ub-activation assay for SPRTN, it was clear that a single substitution in M1-Ub_2_ was defective in stimulating SPRTN auto-cleavage, while the double mutant M1-Ub_2_-I44A/I120A lost most of its ability to stimulate both auto-cleavage and substrate-cleavage activity of SPRTN (Supplementary Fig. S4E and F). We also generated a M1-Ub_6_ variant with mutations in the first two Ub moieties (Fig. 4E and F). As expected, the cleavage kinetics was faster in the presence of wild-type M1-Ub_6_ than in M1-Ub_4_. More pronouncedly, the M1-Ub_6_-I44AI120A mutant was weaker than the M1-Ub_4_ in the activation of SPRTN (Fig. 4E and F), further validating the essential role of the Ub I44 patch in the activation effect.

Altogether, these data suggest that the SPRTN core region possesses the intrinsic ability to bind Ub and that Ub binds to the SPRTN core through the classical I44 hydrophobic patch. Ub binding to the SPRTN core is essential for stimulating SPRTN’s protease activity. Most importantly, the affinity of the SPRTN core for Ub is increased with the length of Ub chains, suggesting that SPRTN binds Ub chains with an avidity effect, consequently leading to SPRTN protease activation.

### The SPRTN core region contains an intrinsic ubiquitin-binding interface

**Figure 5.**
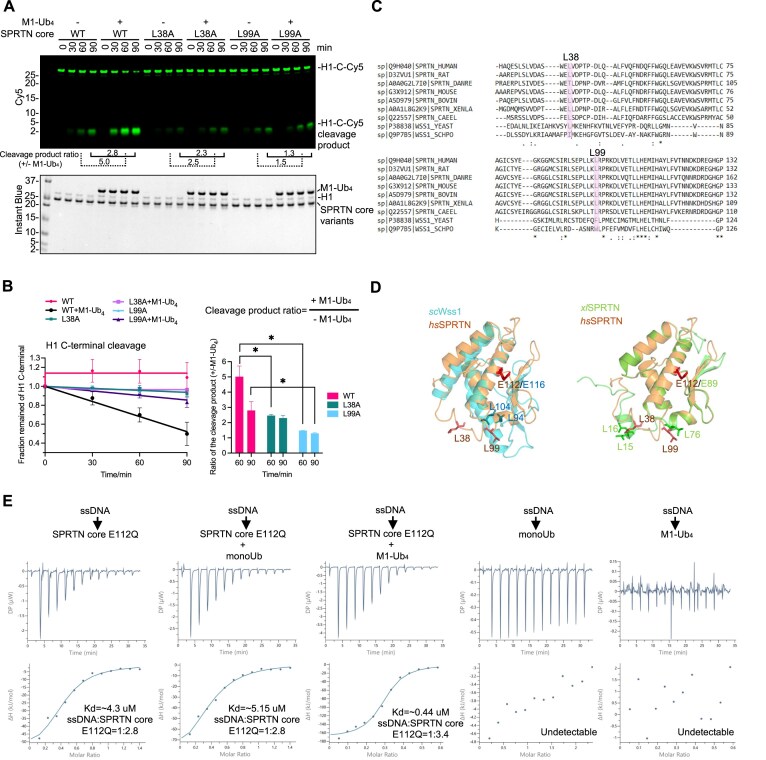
The SPRTN core region of SPRTN contains an intrinsic ubiquitin-binding interface. (**A**) Validation of SPRTN core mutants on the effect of Ub-mediated activation. Recombinant SPRTN core variants (2 μM) and H1-C-Cy5 (1 μM) were incubated in the absence or presence of M1-Ub_4_ (2 μM) in combination with dsDNA_20/23nt (2.7 μM) with the indicated time at 30°C. The reaction was analysed with SDS–PAGE followed by Cy5-scanning on Typhoon FLA 9500 (GE Healthcare) and Instant Blue staining. Representative figure from three repeats. The ratio of cleavage product intensity with/without M1-Ub_4_ at 60 and 90 min for each variant is indicated below the gel. (**B**) Kinetics of the full-length H1-C-Cy5 substrate (C-terminal cleavage rate) and the ratio of cleavage product intensity with/without M1-Ub_4_ at 30 and 90 min, respectively, from Fig. 5A. Significant analysis was performed by unpaired *t*-test (Prism); *n* = 3; error bar, SD; *P < 0.05. (**C**) Multiple protein sequence alignments of representative SPRTN from vertebrates and Wss1 from yeast by CLUSTAL. (**D**) Superimposition of the human SPRTN MPD domain (AlphaFold Protein Structure Database Entry: AF-Q9H040-F1-v4) with the WLM domain from Wss1 (AlphaFold Protein Structure Database Entry: AF-P38838-F1-v4) (left) or MPD domain from Xenopus SPRTN (AlphaFold Protein Structure Database Entry: AF-A0A1L8G2K9-F1-v4) (right). WLM, Wss1-like metalloprotease domain. (**E**) ITC analysis of the binding between ssDNA (20 nt) and SPRTN core (E112Q) in the presence of Ubs (monoUb, M1-Ub_4_, at the equal molar of SPRTN core (E112Q)). The dissociation constant (*K*_d_) and stoichiometry of binding (N) are indicated here and summarized in Supplementary Table S2.

To identify the key residues within the SPRTN core region that engage with the I44 patch of Ub and to have experimental proof that the SPRTN core possesses an intrinsic Ub-binding interface, we carefully looked at the binding interface from the AlphaFold model. We identified L38 and L99 as likely counterparts in the hydrophobic interaction with the I44 patch on Ubiquitin (Fig. 4B). SPRTN core variants bearing the L38A or L99A mutation were generated to validate this prediction. We probed their protease activity by comparing it to the SPRTN core wild-type with or without the M1-Ub_4_ chain in the reaction (Fig. 5A and B; compare the cleavage product ratio indicated below the gel in Fig. 5A). The SPRTN core wild-type protease activity was stimulated by M1-Ub_4_ 5-fold when analysed at 60 min. The L38A and L99A variants of the SPRTN core region only possessed residual protease activity compared to the WT. However, the L99A mutant largely lost the ability to be stimulated by M1-Ub_4_ (only 1.5-fold at 60 min), as demonstrated by the reduced ratio between the cleavage products in the presence/absence of M1-Ub_4_ at individual time points, whereas L38A had a minor effect (2.5-fold at 60 min) on Ub-dependent SPRTN activation (Fig. 5A and B). These data demonstrate the important role of the two residues in the ubiquitin-mediated activation effect.

Sequence alignment also indicated that residues L38 and L99 are highly conserved in vertebrates (Fig. 5C). Therefore, we named this Ub binding interface the **U**biquitin-binding interface of **S**prT **D**omain (**USD**). USD of human SPRTN could be very well superimposed with the equivalent leucine residues in *Xenopus* SPRTN protein (Fig. 5D). However, the functional homolog of SPRTN in yeast, Wss1, and its WLM protease domain could only partially align with the vertebrate/human USD interface. The region in Wss1 corresponding to the human SprT loop containing L38 is absent (Fig. 5D). Altogether, our results demonstrate the identification of a Ub-binding interface, the USD, in the SPRTN core region as the essential module for the rapid activation of SPRTN protease in vertebrates.

As the SPRTN core carries both DNA-binding and Ub-binding properties, we used a simplified model to address the ternary complex consisting of DNA, ubiquitin, and SPRTN core. We investigated the binding stoichiometry of ssDNA: SPRTN core in the presence of monoUb or M1-Ub_4_ by ITC (Fig. 5E and Supplementary Table S2). Notably, the binding stoichiometry of ssDNA: SPRTN core changed from ∼1:2.8 without Ub or with monoUb, to ∼1:3.4 in the presence of equal molar amounts of M1-Ub_4_ and SPRTN core. The addition of an equimolar amount of M1-Ub_4_ and SPRTN core also increased the affinity of SPRTN to ssDNA roughly 10-fold (Fig. 5E and Supplementary Table S2), indicating that the longer Ub chains facilitate the recruitment of SPRTN to DNA.

### Polyubiquitinated substrate rapidly stimulates the SPRTN proteolysis

DPCs are heavily polyubiquitinated, and SPRTN is an essential enzyme for DPC proteolysis repair [34, 38, 50]. Thus, we speculated whether ubiquitinated substrates also stimulate SPRTN protease activity.

**Figure 6.**
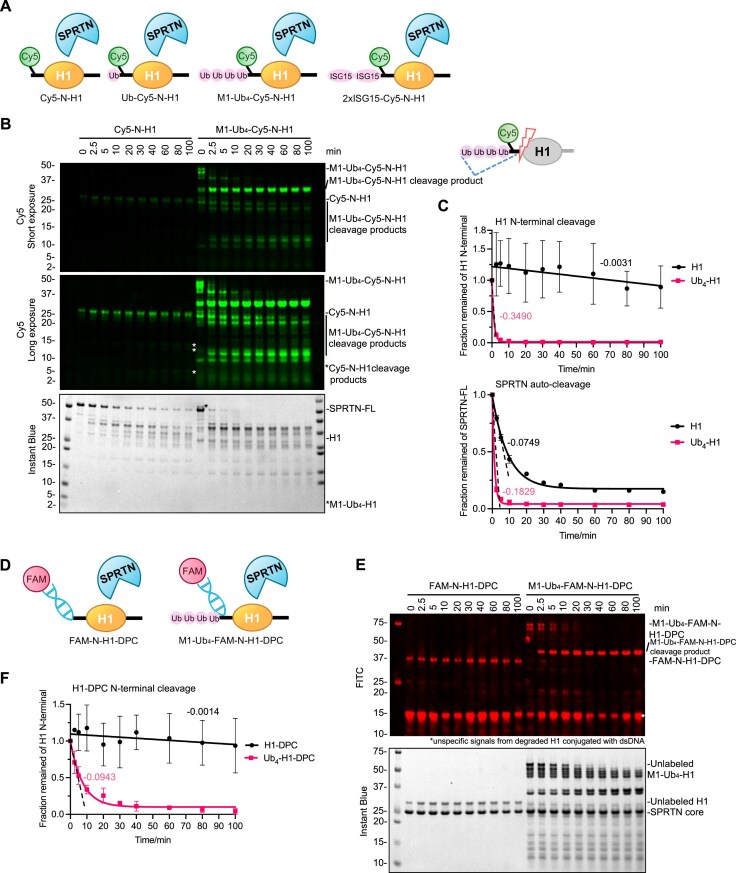
SPRTN rapidly resolves polyubiquitinated DPCs. (**A**) Design of the defined H1 substrates with post-translational modifications for SPRTN cleavage assay (Cy5-labelled all on H1 N-terminal). All the constructs have a His-tag on the N-terminal. (**B**) SPRTN cleavage assay towards Cy5-N-H1 and M1-Ub_4_-Cy5-N-H1. Recombinant full-length SPRTN (2 μM) and H1 substrates (1 μM) were incubated in the presence of dsDNA_20/23nt (2.7 μM) with the indicated time at 30°C. The reactions were analysed by SDS–PAGE followed by Cy5-scanning on an iBright 1500 imaging system (Invitrogen) and Instant Blue staining. Representative figure from two repeats. (**C**) Kinetics of the full-length H1 substrates (N-terminal cleavage rate) and the full-length SPRTN (auto-cleavage rate) from Fig. 6B. Cy5 and SPRTN-FL signals were analysed by the iBright Analysis Software (Invitrogen). For H1 N-terminal cleavage, the kinetic data for H1 were fitted with simple linear regression (Prism), while the first 2.5 min data points for Ub_4_-H1 were fitted with simple linear regression (Prism). The kinetic data for the whole time course of Ub_4_-H1 were fitted with one phase exponential decay-least squares fit (Prism). For SPRTN auto-cleavage, the kinetic data for the whole time course were fitted with one phase exponential decay-least squares fit (Prism). The kinetic data for the first 5 min were fitted with simple linear regression (Prism). All the velocity values for the linear fitting are indicated next to the corresponding dash lines; *n* = 2; error bar, SD. (**D**) Design of the FAM-dsDNA labelled H1-DPC substrates (FAM-N-H1-DPC and M1-Ub_4_-FAM-N-H1-DPC) for SPRTN cleavage assay. (**E**) SPRTN core cleavage assay towards the defined H1-DPC substrates from Fig. 6D. Recombinant SPRTN core (2 μM) and H1-DPC substrates (∼0.1 μM) were incubated at the indicated time at 30°C. The reaction was analysed by SDS–PAGE followed by FITC-scanning on an iBright 1500 imaging system (Invitrogen) and Instant Blue staining. Representative figure from three repeats. (**F**) Kinetics of the full-length H1-DPC substrates (N-terminal cleavage rate) from Fig. 6E. FITC signals were analysed by the iBright Analysis Software (Invitrogen). Kinetic data for H1-DPC were fitted with simple linear regression (Prism) with the velocity value indicated. Kinetic data for Ub_4_-H1-DPC were fitted with one phase exponential decay-least squares fit (Prism). The first 5 min data points from Ub_4_-H1-DPC were fitted with simple linear regression (Prism) with the velocity indicated next to the dashed line; *n* = 3; error bar, SD. See also Supplementary Fig. S5.

To this end, we employed several histone H1 substrates where monoUb, M1-Ub_2,_ M1-Ub_4_, or ISG15 were fused to the N-terminus of Cy5-labelled Histone H1 (Cy5-N-H1) for SPRTN proteolysis (Fig. 6A). Firstly, SPRTN cleavage kinetics was monitored for the unmodified Cy5-N-H1 and M1-Ub_4_-fused H1 (M1-Ub_4_-Cy5-N-H1). Surprisingly, SPRTN cleavage towards M1-Ub_4_-Cy5-N-H1 was enormously accelerated (<2.5 min) (Fig. 6B and C) compared to the simple addition of free M1-Ub_4_ chains in the reaction (>60 min; Fig. 2E and F). The linear fitting for the whole time course from Cy5-N-H1 and the first 2.5 min from M1-Ub_4_-Cy5-N-H1 was compared at a difference of ∼110-fold change, suggesting that Ub chains attached to the substrate stimulate SPRTN proteolytic activity more potently than free Ub chains (Fig. 6B and C). However, this effect was not so pronounced on SPRTN auto-cleavage (Fig. 6B and C; bottom panel; ∼2.4-fold change; data from the first 5 min for both conditions was linearly fitted). This result was also achieved with the SPRTN core region (26–240 aa; this truncated version of SPRTN does not have a pronounced autocleavage effect) when analysed for histone H1 cleavage (Supplementary Fig. S5A and B). Moreover, the rapid cleavage of Cy5-Ub_4_-N-H1 by the SPRTN core region was compromised by the L38A and L99A mutations, albeit the L38A exhibited a milder phenotype (Supplementary Fig. S5C and D).

Notably, a major cleavage product between 25 and 37 kDa from M1-Ub_4_-Cy5-N-H1 accumulated over time (Fig. 6B and Supplementary Fig. S5A). This product retains the N-terminus of H1 (as it was Cy5-visible) and full-length M1-Ub_4_ (as it was detected with an anti-His antibody) (Supplementary Fig. S5E), indicating that SPRTN did not cleave within the Ub chains. Similar to unmodified H1 (Cy5-N-H1), the cleavage kinetics of mono-ubiquitinated H1 (Ub-Cy5-N-H1) or H1 fused to two tandem molecules of ISG15 (2xISG15-Cy5-N-H1), which mimic the structure of M1-Ub_4_, was not as fast as M1-Ub_4_-Cy5-N-H1 (Supplementary Fig. S5F), in line with our previous observations (see Fig. 2E and G).

Histone H1 fused with M1-Ub_4_ was conjugated to dsDNA to mimic a modified DPC (M1-Ub_4_-FAM-N-H1-DPC) (Fig. 6D). Similar to unconjugated M1-Ub_4_-N-H1, ubiquitinated FAM-N-H1-DPC was a better SPRTN substrate than its non-ubiquitinated version (Fig. 6E and F). Linear fitting for the whole time course from FAM-N-H1-DPC and the first 5 min from M1-Ub_4_-FAM-N-H1-DPC was compared to better understand the rate difference. It clearly showed that the SPRTN core cleaves the ubiquitinated H1-DPC roughly 67-fold faster than the non-ubiquitinated H1-DPC (Fig. 6F). These results collectively indicate that SPRTN proteolysis is greatly activated through polyubiquitinated DPC substrates.

### USD domain binds ubiquitin chains by avidity, and the UBZ domain by high affinity

Although we have demonstrated that the UBZ domain of SPRTN is not involved in the ubiquitin-activation effect of SPRTN proteolytic activity (Fig. 3A and B), UBZ still has vital roles in regulating SPRTN by its Ub-binding ability and substrate recruitment [34, 47–49]. As both the USD and the UBZ domains interact with Ub, we asked if the two domains compete for ubiquitin binding.

**Figure 7.**
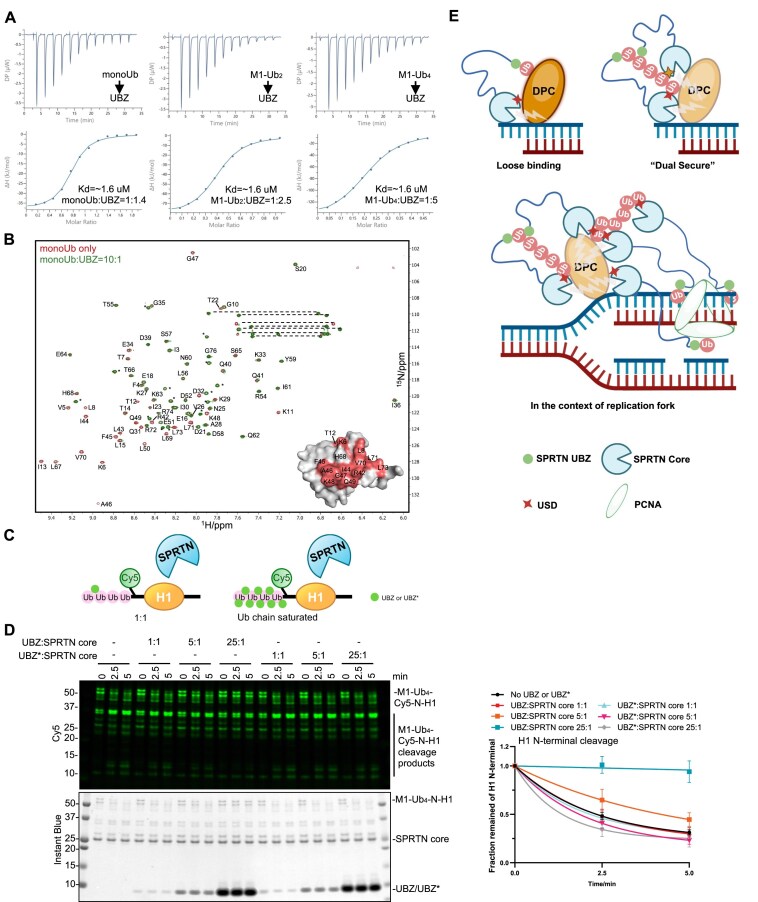
SPRTN UBZ and SPRTN core work in concert to resolve polyubiquitinated DPC substrates. (**A**) ITC analysis of the binding between SPRTN UBZ domain and Ubs (monoUb, M1-Ub_2_, M1-Ub_4_). The dissociation constant (*K*_d_) and stoichiometry of binding (N) are indicated here and summarised in Supplementary Table S3. (**B**) Superimposition of the 950 MHz ^1^H-^15^N HSQC spectra of monoUb alone (in red contours) or with the addition of SPRTN UBZ domain at a molar ratio of 10:1 (in green contours). Spectra were plotted using the MestReNova software. Residues with complete broadening are highlighted in salmon on the monoUb surface (PDB: 1UBQ). The signals were assigned according to previously reported chemical shifts of ubiquitin (BMRB entry: 17769). *Signals from the N-terminal His tag on monoUb. (**C**) Design of the Ub-binding competition between SPRTN core and SPRTN UBZ domain. (**D**) Ub-binding competition between SPRTN core and SPRTN UBZ or UBZ* (C456AC459A). Recombinant SPRTN core (2 μM) and M1-Ub_4_-Cy5-N-H1 (1 μM) were incubated in the presence of dsDNA_20/23nt (2.7 μM) in different ratios of UBZ:SPRTN core from 1:1 to 25:1 with the indicated time at 30°C. The reaction was analysed with SDS–PAGE followed by Cy5-scanning on an iBright 1500 imaging system (Invitrogen) and Instant Blue staining. Right panel: Kinetics of the full-length M1-Ub_4_-Cy5-N-H1 (N-terminal cleavage rate). Kinetic data were fitted with one phase exponential decay-least squares fit (Prism); *n* = 3; error bar, SD. (**E**) Models of SPRTN UBZ and SPRTN core working in concert to resolve ubiquitinated DPC substrates. See also Supplementary Fig. S6.

To this end, we probed the affinity between the UBZ domain (452–489 aa) and monoUb, M1-Ub_2_ or M1-Ub_4_. ITC clearly showed a similar affinity of UBZ, but not its Ub-binding deficient variant UBZ* (C456AC459A), to monoUb or Ub chains at ∼ 1.6 μM (Fig. 7A, and Supplementary Fig. S6A and Supplementary Table S3), which was much higher than the affinity between SPRTN core region and monoUb or Ub chains (Fig. 4A and Supplementary Fig. S4A). We also tested UBZ and UBZ* domains by size-exclusion chromatography with multi-angle light scattering (SEC-MALS), which confirmed that they stay in a monomeric state (Supplementary Fig. S6B and Supplementary Table S4). The similar affinity of UBZ to monoUb and Ub chains and the monomeric state suggested that the UBZ domain does not bind to Ub in an avidity manner. Interestingly, according to the AlphaFold prediction of the interaction between the UBZ domain and monoUb, UBZ also interacts mainly with the ubiquitin I44 patch (Supplementary Fig. S6C). To further confirm this, the perturbed surface on ubiquitin upon UBZ titration was also probed by NMR (Fig. 7B and Supplementary Fig. S6D). It largely overlapped with the surface identified in the NMR profile of monoUb upon SPRTN core titration (Fig. 4C), indicating the possibility that UBZ and SPRTN core (via USD) compete to bind ubiquitin at the same position. To investigate the possible competition effect, the UBZ or UBZ* domain was titrated into a reaction where M1-Ub_4_-Cy5-N-H1 was used as the substrate for SPRTN core proteolytic cleavage (Fig. 7C). Interestingly, UBZ, but not UBZ*, only inhibited SPRTN core protease activity when titrated in significant excess (molar ratio UBZ:SPRTN core = 25:1) but not in the equimolar ratio (1:1) (Fig. 7D). This suggests that if the SPRTN UBZ domain engages with polyubiquitin chains on the substrate, the SPRTN core region can still be accommodated to the same chain. This also aligns with our data where both full-length SPRTN and SPRTN core could rapidly resolve ubiquitinated H1 with similar kinetics (Fig. 6B and C, and Supplementary Fig. S5A and B).

The higher affinity between UBZ and Ub (1.6 μM) supports the idea that UBZ is the general Ub sensor. On the other hand, the avidity effect between the SPRTN core and Ub chains (from ∼770 μM for monoUb to ∼35 μM for M1-Ub_6_) and the ability of longer ubiquitin chains to efficiently stimulate SPRTN protease activity suggest that ubiquitin chain is another key factor in bringing SPRTN close to the DPC substrates, in addition to DNA. These results demonstrate that the N-terminally located SPRTN core (with its USD interface) and the C-terminally located UBZ domain work together rather than compete to resolve polyubiquitinated substrates/DPCs.

## Discussion

Replication-coupled DPC proteolysis repair is an essential DNA repair mechanism for genome stability and protection from accelerated ageing and cancer [29]. SPRTN protease emerged as the crucial enzyme for this repair. However, the mechanism by which SPRTN achieves specificity for DPC proteolysis, instead of targeting the replisome, remains enigmatic. Interestingly, DPC and DPC-like proteins are heavily polyubiquitinated by various E3-ubiquitin ligases, especially in response to chemotherapy drugs or formaldehyde [8, 27, 34, 40, 50–52], but whether and how polyubiquitinated substrates/DPCs regulate SPRTN proteolysis has not yet been elucidated.

The work presented here demonstrates that SPRTN is a specialized protease localized to and rapidly activated by polyubiquitinated chains on the substrates/DPCs using its intrinsic ubiquitin-binding modules, UBZ and USD, respectively. SPRTN cleaves the polyubiquitinated substrates irrespective of the chain linkages, making SPRTN a universal DPC protease for polyubiquitinated DPCs that can occur regardless of the E3-ubiquitin ligase involved in DPC repair. This work clarifies the essential question in the field: How does the SPRTN protease specifically process DPCs and thus minimize the cleavage of other replicative proteins such as PCNA?

### Polyubiquitination of DPCs governs SPRTN’s specificity and its rapid proteolysis

SPRTN was identified as a protein that contains a UBZ domain that regulates PCNA mono-ubiquitination status during translesion DNA synthesis and a cofactor of the Ub-dependent ATPase p97 (also known as VCP), the central component of the Ub system [47–49, 53, 54]. However, how Ub or the ubiquitination of substrates/DPCs regulates SPRTN’s protease activity and specificity is enigmatic. There is a consensus in the field that the UBZ domain, by binding to monoUb or polyUb chains, regulates SPRTN stability and its recruitment to DPCs, respectively [32, 38]. Still, this does not clarify how its enzymatic activity and substrate specificity are controlled.

It is evident that many factors stimulate SPRTN proteolysis and cellular response to DPC-inducing agents [20, 24, 25, 48, 49, 53–58], but how the polyubiquitination of substrates/DPCs, the robust post-translational signal on DPCs induced by formaldehyde, chemotherapy drug treatment or even during covalent attachment of HMCES to abasic sites on ssDNA [8, 34, 50, 52] regulates SPRTN proteolysis is not known. One model is that when the replicative helicase CMG approaches DPCs, it bypasses the DPC lesion but is uncoupled from the PCNA–DNA polymerase complex, thus forming a single-strand DNA structure where DPCs are attached [33]. When the PCNA–DNA polymerase complex approaches the DPC, this is a perfect DNA structure (ss/dsDNA junction) for SPRTN activation [3]. However, even though this ss/dsDNA junction is indeed the best DNA substrate for SPRTN proteolysis, and we confirm it too (Fig. 1), it does not explain how SPRTN gains specificity between DPCs and replication machinery. Moreover, this ss/dsDNA structure also takes hours to activate SPRTN proteolysis *in vitro* [3, 19, 31].

Specifically, we identified polyubiquitination of DPC as the primary signal driving SPRTN’s specificity and facilitating the rapid proteolysis of DPCs. SPRTN, in addition to its DNA-binding activation mode (the first tier of activation), is also an Ub-activated protease (the second tier of activation) with a previously unidentified Ub-binding interface (USD) in its catalytic core domain. USD barely detects monoUb (affinity for mono Ub is ∼770 μM, Supplementary Fig. S4C) but rapidly increases its affinity in an avidity mode for the growing ubiquitin chains (polyubiquitination; USD affinity to M1-Ub_4_ at ∼70 μM, and further increased to ∼35 μM with M1-Ub_6_, Fig. 4A and Supplementary Fig. S4A and Supplementary Table S1). Together with the DNA-binding ability from the ZBD and BR domain, this Ub-threshold property from USD largely defines the substrate selectivity and the timely enzymatic efficiency of SPRTN. In contrast to USD, the C-terminal UBZ domain has a strong affinity for both monoUb and Ub chains (∼1.6 μM, Fig. 7A and Supplementary Table S3), but not outcompetes USD when binding to longer ubiquitin chains (Fig. 7D). Based on our results, we propose a model where two intrinsic Ub-binding modules of SPRTN, USD and UBZ, regulate the spatiotemporal and rapid proteolysis of polyubiquitinated DPCs in a “dual secure” mode (Fig. 7E).

### SPRTN auto-cleavage versus DPC proteolysis

It is also evident that SPRTN auto-cleavage is strongly induced by Ub chains, raising the question of how SPRTN auto-cleavage and substrate-cleavage are regulated.

It was previously shown that SPRTN still retains DNA-binding property and partial activity towards the substrate upon auto-cleavage [22]. SPRTN’s residual activity has been previously characterized, where the major auto-cleaved leftover of SPRTN (called SPRTN-auto hereafter) could still process H1 in the presence of ssDNA, at a similar level of SPRTN-ΔC, though weaker than full-length SPRTN [22]. Therefore, SPRTN protease activity is not entirely destroyed by auto-cleavage. In addition, as SPRTN-auto retains the catalytic SprT domain, where the USD is located, SPRTN-auto can likely still be activated by Ub chains. This could also explain our findings that H1 can still be processed at a later stage (30–60 min) in the presence of M1-Ub_4_ when full-length SPRTN is almost completely auto-cleaved (Fig. 2D and F). Therefore, SPRTN-auto can be partially recycled during the processing of polyubiquitinated DPCs.

In terms of regulating both SPRTN auto-cleavage and DPC cleavage, we hypothesize that upon SPRTN recruitment to polyubiquitinated DPCs via the UBZ domain (which has a high affinity for Ub chains compared to the USD; see Fig. 7A versus Fig. 4A), autocleavage occurs near the DNA as SPRTN attacks the substrates, allowing the release of the catalytic module (SPRTN-auto) from full-length SPRTN. Even without the UBZ domain (the general Ub sensor), SPRTN-auto still possesses affinity to polyubiquitin chains due to its intact USD and can thus process polyubiquitinated DPCs, very likely the same ones that have started to be cleaved by full-length SPRTN. Since the UBZ and other signalling modules are absent, SPRTN is unlikely to regain full functionality. Therefore, it will most likely be ultimately degraded.

### A model of SPRTN specificity at/around the DNA replication fork

SPRTN binds PCNA over its PIP box and is associated with DNA replicative polymerases [25, 47–49, 54, 59]. Uncontrolled SPRTN protease activity at/around the DNA replication fork can be detrimental to cells, as it has been believed that SPRTN is an unspecific protease. Our model suggests that SPRTN can be equally recruited to monoubiquitinated PCNA via its PIP box and UBZ domain. Binding data from SPR also indicates that the SPRTN PIP-UBZ module has a decent affinity to PCNA and monoUb-PCNA (Supplementary Fig. S6E and Supplementary Table S5). As PCNA is a homotrimer and each monomer contains one mono-ubiquitinated site at lysine 164, it is reasonable to speculate that one molecule of monoUb-PCNA homotrimer can accommodate three molecules of SPRTN by binding to both the PIP box and UBZ domain (Fig. 7E). Once the SPRTN C-terminal tail is stabilized on PCNA by the PIP box and UBZ domain, USD allows the recruitment of SPRTN protease core to the sites of polyubiquitinated DPCs either during the ongoing DNA replication (non-ubiquitinated PCNA) or during stalled DNA replication fork (monoUb-PCNA) (Fig. 7E). If the DNA replication fork approaches DPCs, and the DPCs are heavily polyubiquitinated, SPRTN rapidly increases its affinity and specificity towards the growing ubiquitin chains and, consequently, DPCs.

On the other hand, the Ub-threshold of USD could minimize the degradation of PCNA or monoUb-PCNA or DNA polymerases nearby, as these proteins are not heavily polyubiquitinated during unperturbed DNA replication fork progression [60–65]. Our *in vitro* assay demonstrates that SPRTN proteolysis of PCNA or monoUb-PCNA is barely detectable, if at all, even after 4 h (Supplementary Fig. S6F). Moreover, our data are consistent with the previous report, which showed that SPRTN cannot cleave unmodified components of the replisome, such as RFC, RPA, PCNA, or monoUb-PCNA during 2 h of incubation [66]. As SPRTN possesses a large flexible region between the N-terminal USD-containing protease domain and the C-terminal UBZ domain, indicated by AlphaFold prediction (Supplementary Fig. S6G, AlphaFold Protein Structure Database Entry: AF-Q9H040-F1-v4), this also suggests that the catalytic domain of SPRTN could reach polyubiquitinated substrates/DPCs around PCNA–DNA replication while still being attached to PCNA. Our model indicates that SPRTN facilitates the rapid proteolysis of polyubiquitinated substrates/DPCs with high selectivity around the replisome, but the replisome itself is not degraded during unperturbed or translesional DNA synthesis, as its components are not heavily polyubiquitinated. A similar scenario can occur at post-replicative gaps containing abasic sites that are protected by the covalent attachment of HMCES, which is subsequently polyubiquitinated by the E3 ubiquitin ligase RFWD3 and degraded in a proteasome-independent but SPRTN-dependent manner [40].

### SPRTN versus 26S proteasome in the processing of polyubiquitinated DPCs

The K48-linked poly-ubiquitination of proteins is the main ubiquitin signal that governs substrate degradation by the 26S proteasome [67]. On the contrary, SPRTN protease was previously identified to cleave its substrates in a ubiquitination-independent manner *in vitro* [3, 19, 22, 25]. Based on these facts, there is a general opinion in the field that the 26S proteasome is recruited to polyubiquitinated chains on DPCs and thus induces DPC proteolysis. In contrast, SPRTN does not need the ubiquitin chains on DPCs and its proteolysis is activated by the nascent DNA approaching DPCs on the maternal DNA strand (ss/dsDNA junction) [3, 27, 29, 40]. However, it is puzzling that SPRTN proteolysis of DPCs is still dependent on the UBZ domain, and it was suggested that SPRTN is recruited by a ubiquitinated protein around DPC, but other than the DPC itself [27].

Our work indicates that the polyUb signal on the substrates/DPCs is the main catalyst for SPRTN proteolysis. Without the polyubiquitination of substrates/DPCs, SPRTN is an inefficient enzyme that takes hours to cleave its substrates, which contradicts its role in the rapid resolution of DPCs during DNA synthesis. By recognizing the polyUb chains attached to substrates/DPCs, SPRTN becomes a rapid and processive protease that cleaves its substrates within a few minutes (2.5–10 min, Fig. 6). Since SPRTN activation by polyubiquitin chains is not linkage-specific, unlike the proteasome, we propose that SPRTN serves as a universal protease for removing ubiquitinated DPCs, regardless of which E3 ubiquitin ligase is involved in DPC polyubiquitination. This characteristic provides a broad spectrum of substrates/DPCs for SPRTN proteolysis.

To our knowledge, this is the first endopeptidase known to recognize and proteolyze substrates tagged with all tested ubiquitin chains—both homotypic and heterotypic—and whose proteolytic activity increases with the length of the ubiquitin chains. Intriguingly, the proteasomal shuttling factor Ddi1 was recently identified in yeast as a ubiquitin-dependent protease. Ddi1 specifically binds ubiquitin and cleaves polyubiquitinated proteins, targeting those tagged with long ubiquitin chains (>8 ubiquitins) that are K48- or K63-linked [68]. Furthermore, Ddi1 has been identified, along with Wss1, as an essential factor for DPC proteolysis in yeast [69]. Since the yeast orthologue of SPRTN, Wss1, binds SUMO instead of ubiquitin, it is tempting to speculate that yeast Ddi1 serves as the primary protease for the proteolysis of polyubiquitinated DPCs in yeast. This role becomes particularly crucial in SUMO- and Wss1-compromised cells, where Ddi1 becomes essential for yeast cell survival in response to Top1-cc lesions [70]. Thus, we speculate that Ddi1 mimics SPRTN’s proteolytic specificity for polyubiquitinated DPCs in yeast.

Our work proposes that the polyubiquitin chains on DPCs serve as an excellent fine-tuning signal to regulate SPRTN selectivity and proteolysis towards DPCs, thus avoiding unspecific cleavage of non-ubiquitinated replication-associated proteins during DNA synthesis. Indeed, we experimentally demonstrated that PCNA and monoUb-PCNA are barely cleaved by SPRTN (Supplementary Fig. S6F), further supporting our model that polyubiquitin chains on DPCs govern SPRTN’s specificity towards DPC proteolysis.

### The role of USD in SPRTN proteolysis *in vivo*

Our study was conducted *in vitro* using purified proteins, various DNA templates, and histone H1 as a model substrate. The question remains whether our findings are relevant *in vivo*. Encouragingly, the parallel study conducted by the Stingele group also discovered similar results, showing that polyUb chains mediate rapid SPRTN proteolysis [71]. The authors identified and demonstrated that the key residues for binding ubiquitin chains in the SprT domain (L38 and L99) stimulate SPRTN’s ubiquitin-dependent proteolysis *in vitro* and are also important *in vivo*. These are the same residues that we identified for stimulating SPRTN’s proteolysis by ubiquitin chains (Figs 4B  and 5A–C).

Specifically, the Stingele group elegantly showed that mutations in the USD (L38 and L99) of the SPRTN-ΔC variant (where the C-terminal domain, including the UBZ domain, is truncated) cause growth arrest, formation of micronuclei, chromosomal bridges, and G2/M cell cycle arrest in mouse cell lines. SPRTN-ΔC is expressed in patients with RJALS, indicating that the USD domain is essential for maintaining genome stability and cell growth in these patients. However, the effects on genome stability and cell growth were not observed when mouse cells expressing full-length SPRTN harbour a single amino acid mutation in either L38 or L99. The double mutant L38/L99 of full-length SPRTN would likely have a more pronounced phenotype; however, this needs to be demonstrated experimentally.

Therefore, further mechanistic work to address the role of the USD and the stimulation of SPRTN’s protease activity by ubiquitin chains *in vivo* is needed. For example, analyses of DNA replication fork progression using DNA fibers, sensitivity of cells to formaldehyde, and resolution of DPCs in SPRTN wild-type versus SPRTN-L38A or SPRTN-L99A variants would be necessary. Addressing these questions in an elegant manner (*e.g*. by using CRISPR–Cas9-induced mutations in the SPRTN gene) will require additional work.

These independent findings from the Stingele group support the relevance of our results at the cellular level, indicating that the USD, and likely the stimulation of SPRTN proteolysis by ubiquitin chains, are essential for protecting against genomic instability in RJALS patients. Further investigation is needed to determine how the USD contributes to the proteolysis of full-length SPRTN *in vivo*.

In summary, we have identified the poly-ubiquitinated chains as the main signal for SPRTN’s rapid activation, and very likely the main signal for SPRTN’s specificity towards DPC proteolysis, as DPCs are heavily poly-ubiquitinated in a cell.

## Supplementary Material

gkaf638_Supplemental_File

## Data Availability

Further information and requests for resources and reagents should be directed to and will be fulfilled upon reasonable request by the Lead Contact, Kristijan Ramadan (kristijan.ramadan@ntu.edu.sg). All plasmids will be deposited on Addgene: https://www.addgene.org/
