# Supplementary material for: The dual ubiquitin binding mode of SPRTN secures rapid spatiotemporal proteolysis of DNA–protein crosslinks
Source: Nucleic Acids Res. 2025 Jul 21;53(13):gkaf638. doi: 10.1093/nar/gkaf638 (PMC12277127; doi:10.1093/nar/gkaf638)
Supplement: gkaf638_Supplemental_File [file gkaf638_supplemental_file.pdf]

Supplementary Data

The dual ubiquitin binding mode of SPRTN secures rapid spatiotemporal proteolysis of DNA-protein crosslinks

Wei Song[1], Yichen Zhao[1], Annamaria Ruggiano[1, 2], Christina Redfield[3], Joseph A Newman[4], Xiaosheng Zhu[1], Marta García Flores[2], Abimael Cruz-Migoni[1], Rebecca Roddan[1], Anna Pérez-Ràfols[5], Peter McHugh[1], Paul R. Elliott[3] and Kristijan Ramadan[1,6,7]

[1]The MRC Weatherall Institute of Molecular Medicine, Department of Oncology, John Radcliffe Hospital, University of Oxford, Oxford, OX3 9DS, UK

[2]Center for Biological Research Margarita Salas (CIB-CSIC), Spanish National Research Council, Madrid, Spain

[3]Department of Biochemistry, University of Oxford, South Parks Road, Oxford, OX1 3QU, UK

[4]Centre for Medicines Discovery, University of Oxford, Oxford, OX3 7FZ, UK

[5]MRC Protein Phosphorylation and Ubiquitylation Unit, Sir James Black Centre, School of Life Sciences, University of Dundee, Dundee, DD1 5EH, UK

[6]Lee Kong Chian School of Medicine (LKCMedicine), Cancer Discovery and Regenerative Medicine Program, Nanyang Technological University, S636921, [illegible]

[7]Lead Contact

*Correspondence to: kristijan.ramadan@ntu.edu.sg

CONTENT OF THE SUPPLEMENTARY DATA:

- Supplementary Figures 1-6
- Description of Supplementary Figures
- Supplementary Tables (Table S1-S5)
- Key Resource Table

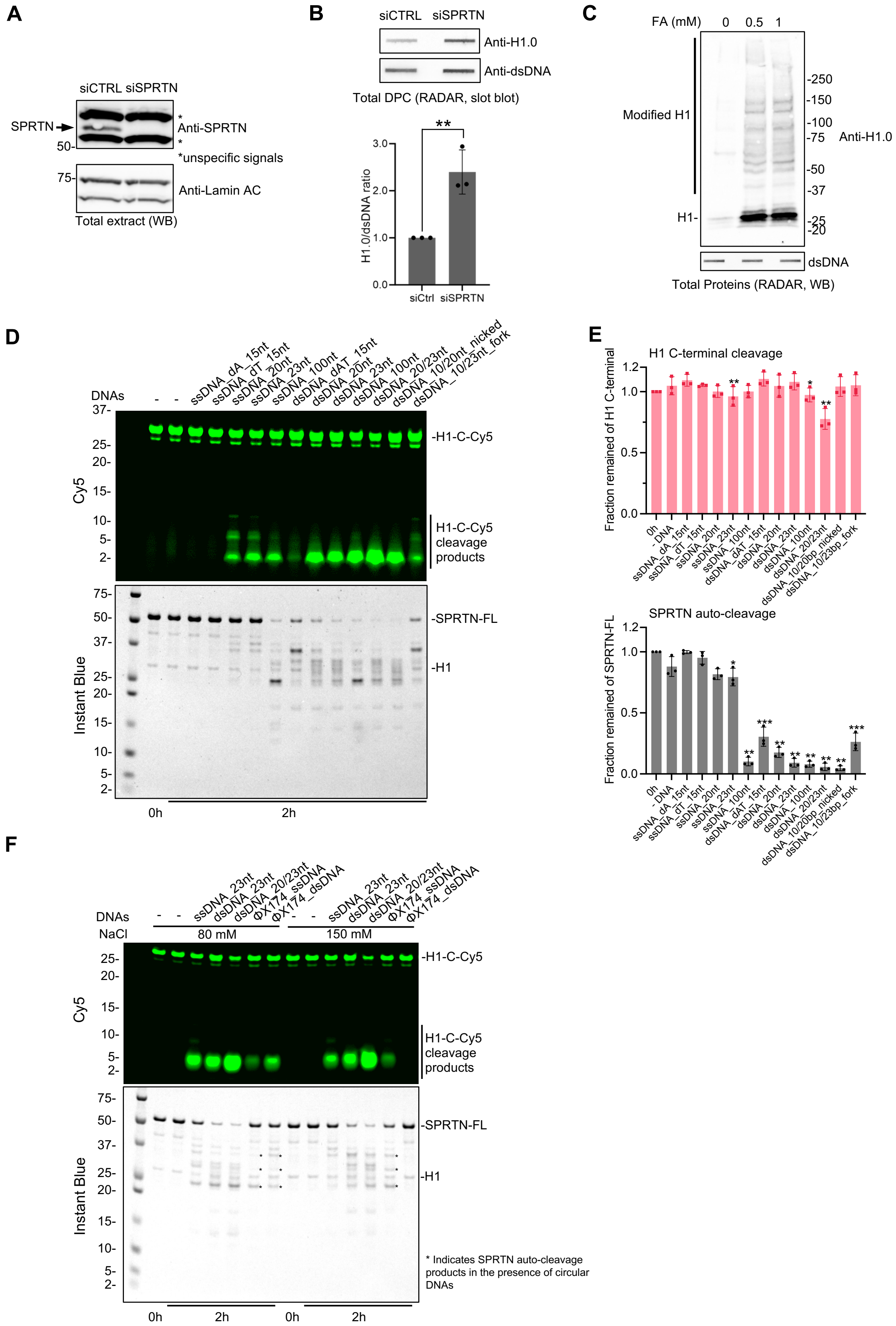

Supplementary Figure 1 (Related to Figure 1)

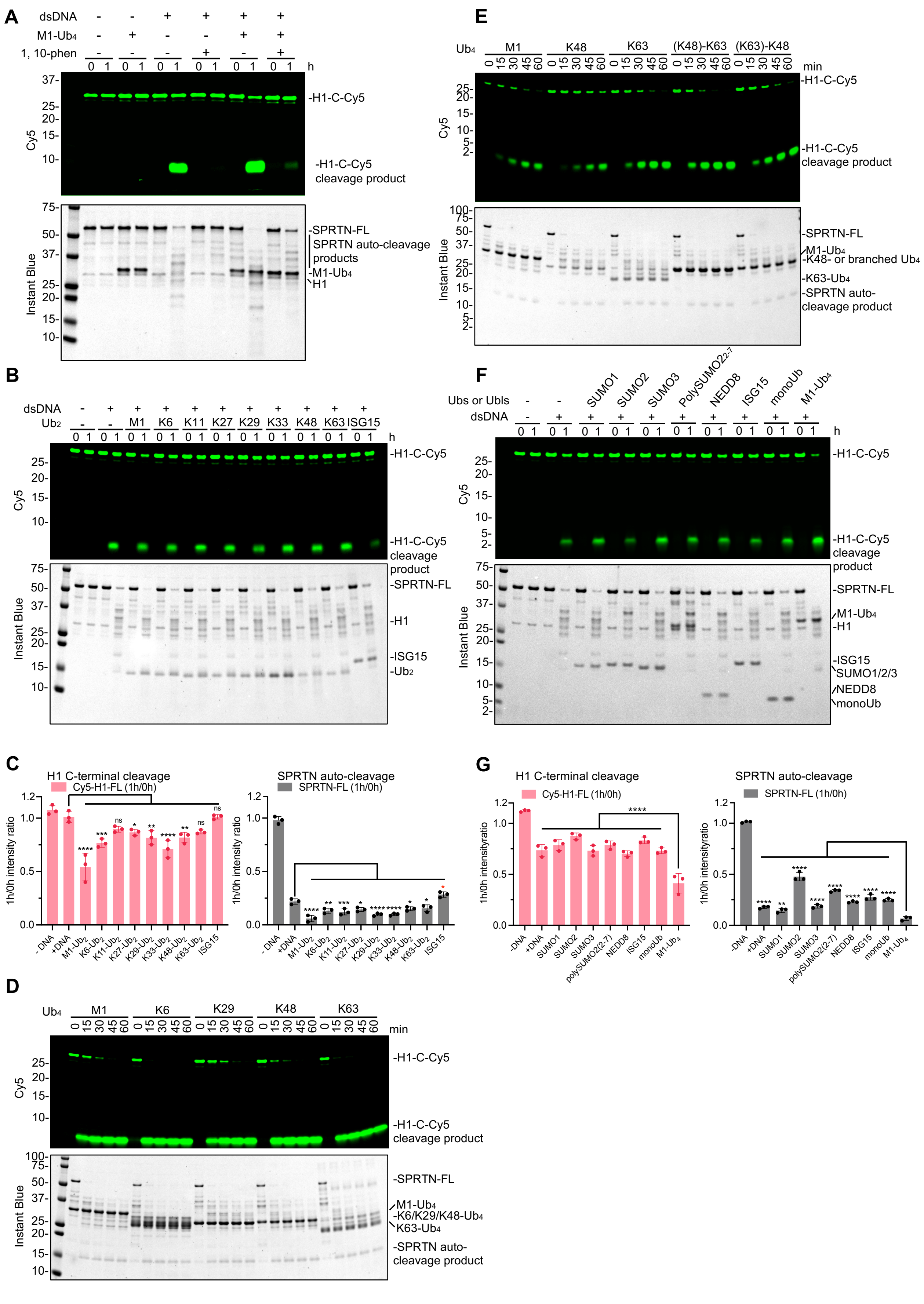

Supplementary Figure 2 (Related to Figure 2)

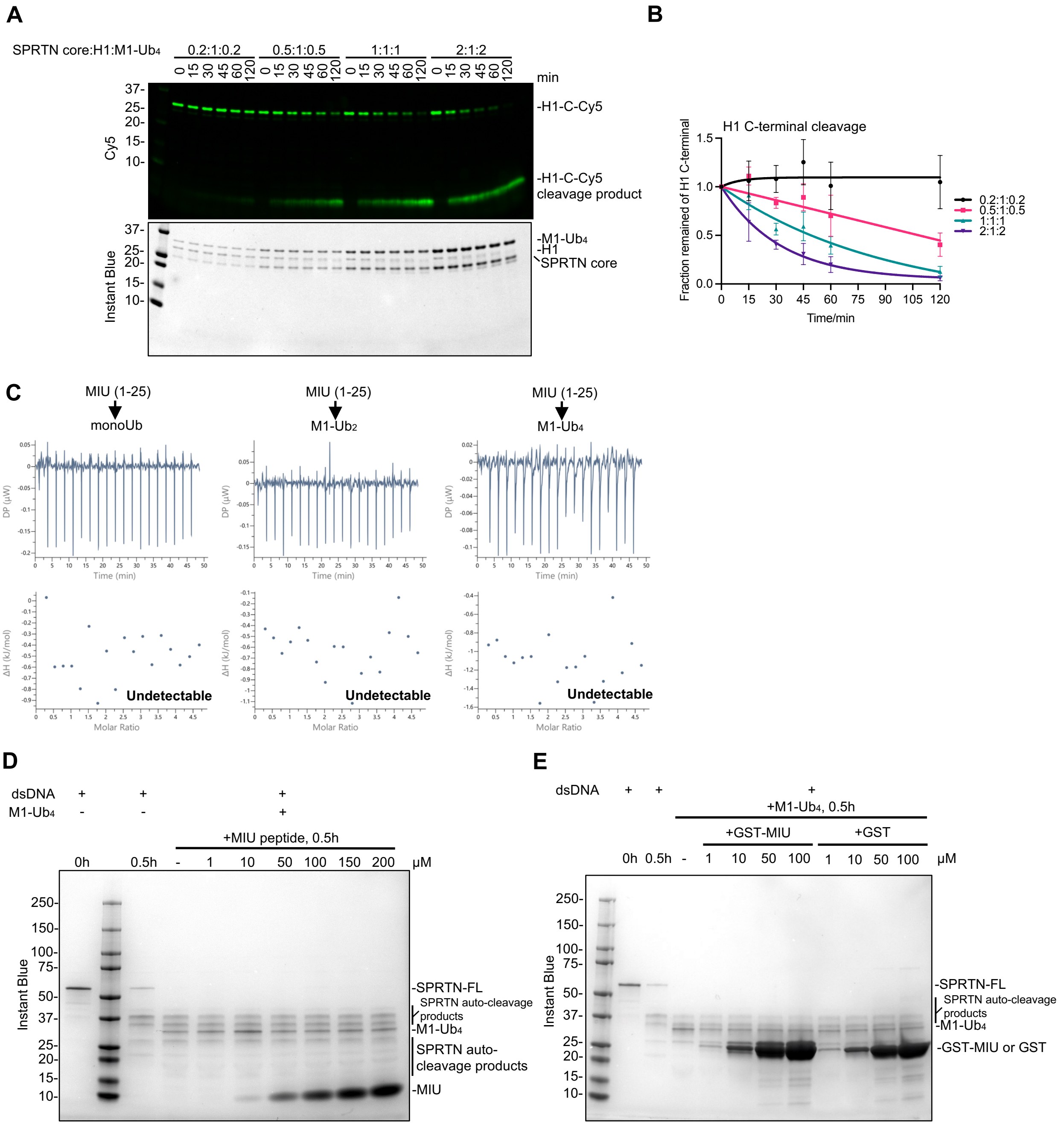

Supplementary Figure 3 (Related to Figure 3)

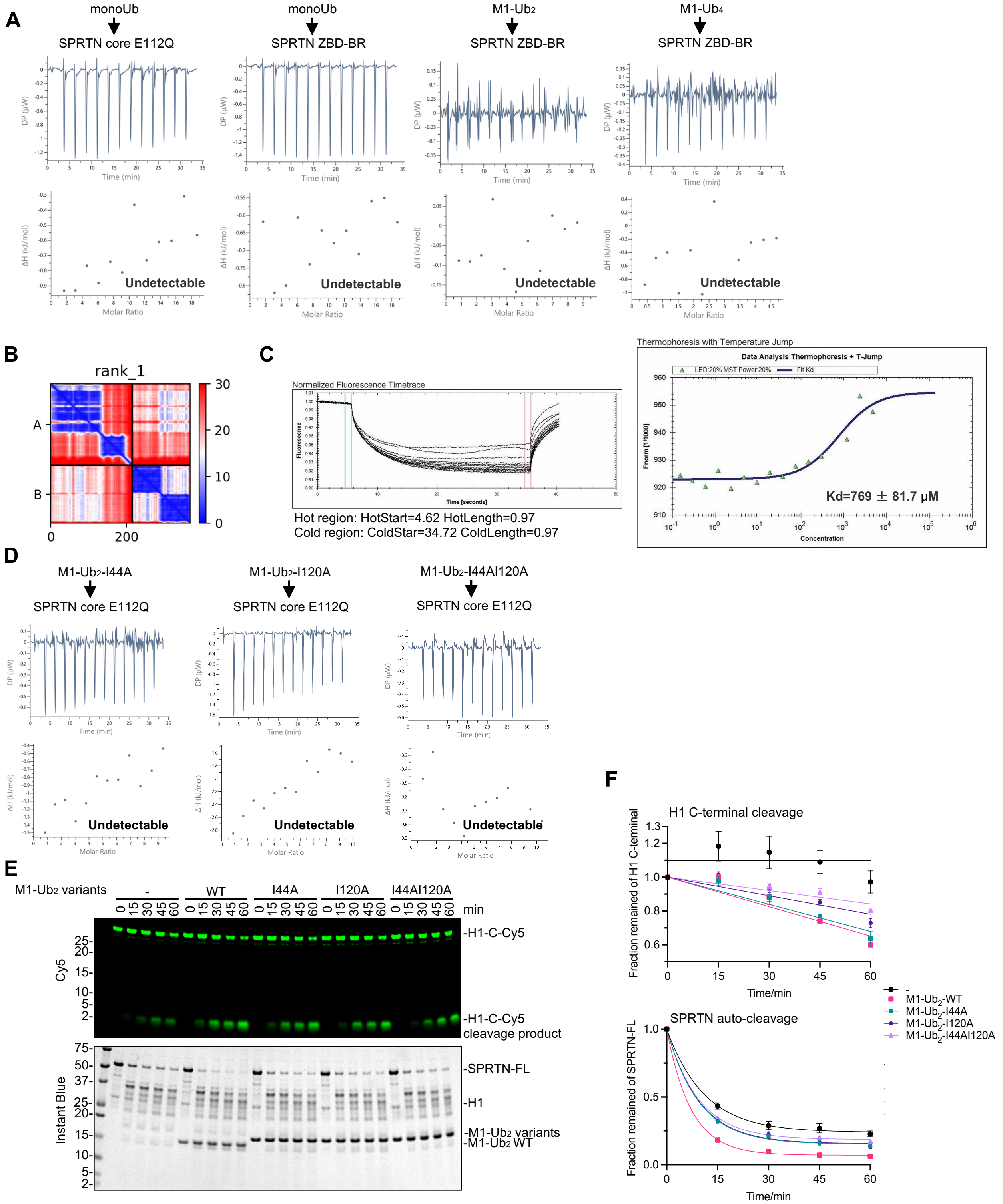

Supplementary Figure 4 (Related to Figure 4)

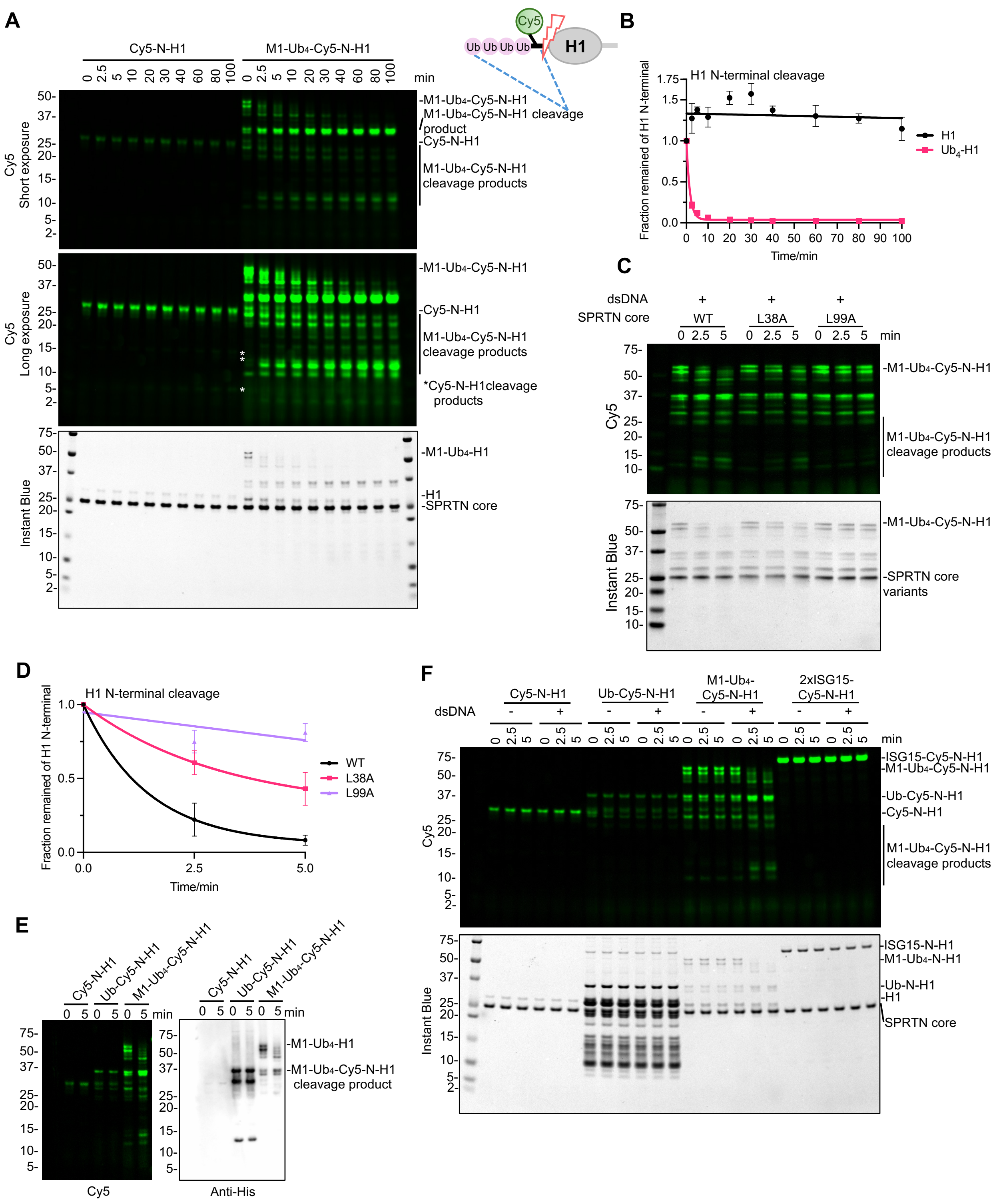

Supplementary Figure 5 (Related to Figure 6)

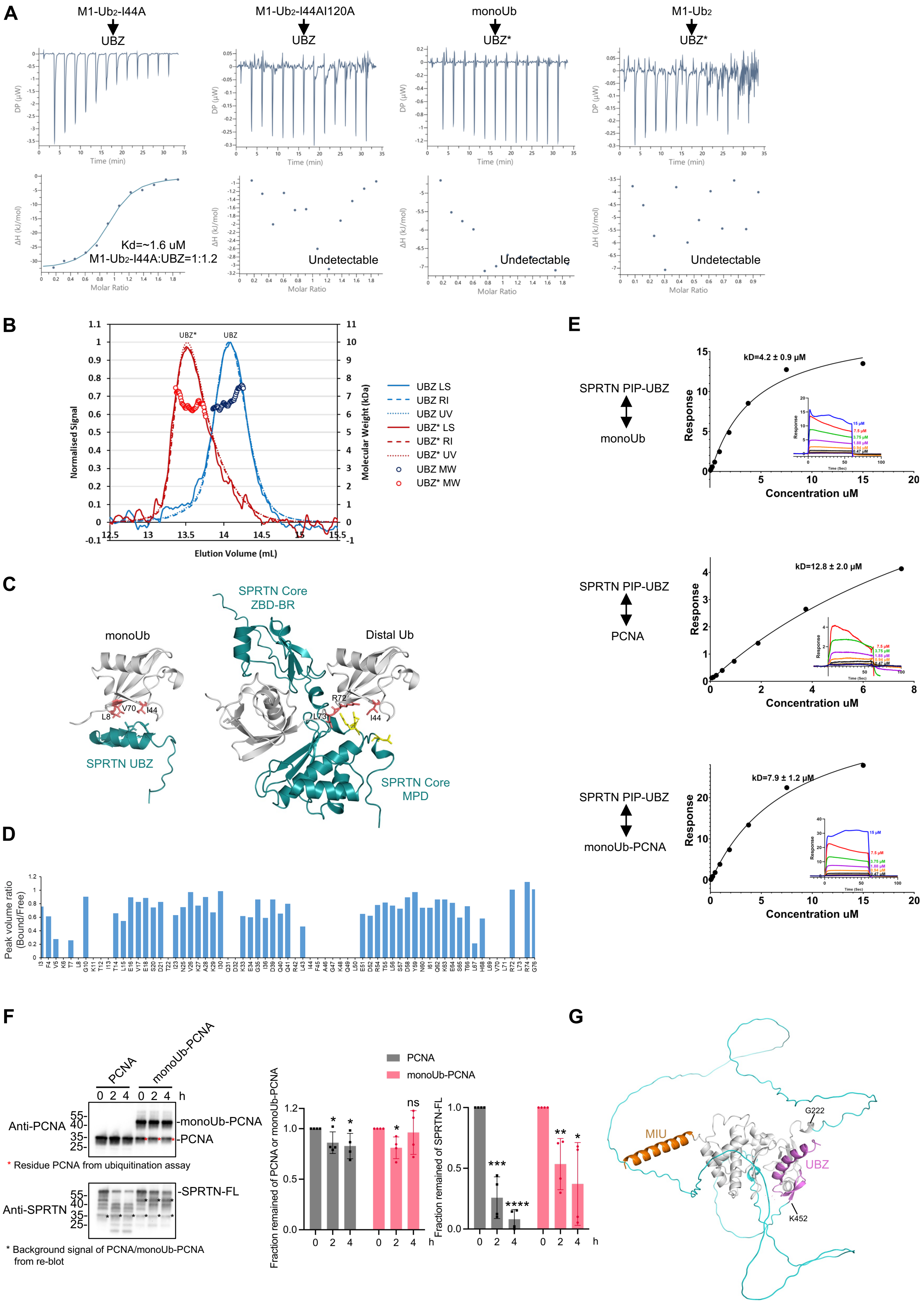

Supplementary Figure 6 (Related to Figure 7)

Supplementary Figures Description

Supplementary Figure 1. SPRTN proteolysis is activated by both ssDNA and dsDNA

(A) Confirmation of SPRTN depletion in U2OS cell line by western blot.

(B) Total DPCs were isolated from the SPRTN-depleted cells (U2OS cell line) by RADAR and analysed by slot blot (upper panel). Representative figure from 3 repeats. Band signals were quantified by Image Lab (Bio-rad). H1.0 signals were normalised against dsDNA (bottom panel). Statistical analysis was performed by unpaired t-test (Prism). n=3. Error bar, SD. **p<0.05.

(C) Total DPCs were isolated from the formaldehyde-treated cells (Hek293 cell line) by RADAR. Total DPCs were analysed by SDS-PAGE followed by western blot against histone H1.

(D) DNA screening for SPRTN cleavage assay. Recombinant full-length SPRTN (2 μM) and H1-C-Cy5 (1 μM) were incubated in the absence or presence of indicated DNA (2.7 uM) for 2h at 30°C. The reaction was analysed by SDS-PAGE followed by Cy5-scanning on Typhoon FLA 9500 (GE Healthcare) and Instant Blue staining. Representative figure from 3 repeats.

(E) Quantification of the signal from the full-length H1-C-Cy5 substrate (C-terminal cleavage rate) and the full-length SPRTN (auto-cleavage rate) from Supplementary Figure 1D. Cy5 signals were analysed by ImageJ. SPRTN-FL signals were analysed by the iBright Analysis Software (Invitrogen). Significant analysis was performed by comparing each data point to "-DNA" by unpaired t-test (Prism) (Data from the 0h time point was excluded from the analysis). n=3. Error bar, SD. *p <0.05; **p <0.005; ***p <0.0005.

(F) SPRTN cleavage assay under 80 mM or 150 mM NaCl conditions in the buffer. Recombinant full-length SPRTN (2 μM) and H1-C-Cy5 (1 μM) were incubated in the absence or presence of indicated DNA (11.5 nM for ss- and ds-circular DNAs; 2.7 uM for the rest of the DNAs; DNA concentration was adjusted to ensure circular DNAs have the equivalent total length to the shorter DNAs) for 2h at 30°C. The reaction was analysed by SDS-PAGE followed by Cy5-scanning on Typhoon FLA 9500 (GE Healthcare) and Instant Blue staining. Representative figure from 3 repeats.

Supplementary Figure 2. Ubs but not Ub-like proteins activate SPRTN proteolysis

(A) SPRTN cleavage assay towards H1-C-Cy5 in the combination of dsDNA_20/23nt with M1-Ub_4 and 1,10-phenanthroline (1,10-phen). Recombinant full-length SPRTN (2 μM) and H1-C-Cy5 (1 μM) were incubated with different combinations of dsDNA_20/23nt (2.7 μM), M1-Ub_4 (2 μM) and 1,10-phenanthroline (0.5 mM) for 1h at 30°C. Representative figure from 3 repeats.

(B) Ub_2 screening for SPRTN cleavage assay. Recombinant full-length SPRTN (2 μM) and H1-C-Cy5 (1 μM) were incubated with indicated Ub_2 or ISG15 (2 μM) in the presence of dsDNA_20/23nt (2.7 μM) for 1h at 30°C. Representative figure from 3 repeats.

(C) Quantification of the signal from the full-length H1-C-Cy5 substrate (C-terminal cleavage rate) and the full-length SPRTN (auto-cleavage rate) from Supplementary Figure 2B. n=3. Error bar, SD.
(D) Ub_4 screening for SPRTN cleavage assay. Recombinant full-length SPRTN (2 μM) and H1-C-Cy5 (1 μM) were incubated with indicated Ub_4 (2 μM) in the presence of dsDNA_20/23nt (2.7 μM) with indicated time at 30°C. Representative figure from 3 repeats.
(E) Branched Ub_4 screening for SPRTN cleavage assay. Recombinant full-length SPRTN (2 μM) and H1-C-Cy5 (1 μM) were incubated with indicated Ub_4 (2 μM) in the presence of dsDNA_20/23nt (2.7 μM) with indicated time at 30°C. Branched Ub_4: (K48)-K63 indicates $[Ub]_2$-^{48,63}Ub-^{63}Ub; (K63)-K48 indicates $[Ub]_2$-^{48,63}Ub-^{48}Ub. Representative figure from 3 repeats.
(F) Ubl screening for SPRTN cleavage assay. Recombinant full-length SPRTN (2 μM) and H1-C-Cy5 (1 μM) were incubated with indicated Ubs or Ubls (2 μM) in the presence of dsDNA_20/23nt (2.7 μM) for 1h at 30°C. Representative figure from 3 repeats.
(G) Quantification of the signal from the full-length H1-C-Cy5 substrate (C-terminal cleavage rate) and the full-length SPRTN (auto-cleavage rate) from Supplementary Figure 2F.
All the reactions were analysed by SDS-PAGE followed by Cy5-scanning on Typhoon FLA 9500 (GE Healthcare) and Instant Blue staining. Cy5 signals were analysed by ImageJ. SPRTN-FL were analysed by the iBright Analysis Software (invitrogen). Significant analysis from Supplementary Figure 2C and S2G was performed by ANOVA (Prism) (Data from "-DNA" was excluded from analysis). *p <0.05; **p <0.005; ***p <0.0005; ns: not significant.

Supplementary Figure 3. SPRTN core region of SPRTN is sufficient for the rapid activation of SPRTN protease mediated by Ub
(A) SPRTN core cleavage assay towards H1-C-Cy5 with M1-Ub_4. Recombinant SPRTN core and H1-C-Cy5 were incubated with M1-Ub_4 with the indicated ratio in the presence of dsDNA_20/23nt (2.7 μM) with the indicated time at 30°C. H1-C-Cy5 was kept constantly at 1 μM in all conditions. The reactions were analysed by SDS-PAGE followed by Cy5-scanning on an iBright 1500 imaging system (Invitrogen) and Instant Blue staining. Representative figure from 3 repeats.
(B) Cleavage kinetics of the signal from the full-length H1-C-Cy5 substrate (C-terminal cleavage rate) from Supplementary Figure 3A. Cy5 and SPRTN-FL signals were analysed by the iBright Analysis Software (Invitrogen). Kinetic data were fitted with one phase exponential decay - least squares fit (Prism). n=3. Error bar, SD.
(C) Isothermal titration calorimetry (ITC) analysis of the binding between MIU peptide (1-25 aa) and Ubs (monoUb, M1-Ub_2, M1-Ub_4).
(D) Competition binding assay of MIU peptide to M1-Ub_4. SPRTN auto-cleavage was monitored. Recombinant full-length SPRTN (2 μM) and M1-Ub_4 (1 μM) were incubated with MIU peptide with the indicated concentration in the presence of dsDNA_20/23nt (2.7 μM) for 0.5h at 30°C. The reaction was analysed by SDS-PAGE.

(E) Competition binding assay of GST-MIU to M1-Ub_4. SPRTN auto-cleavage was monitored. Recombinant full-length SPRTN (2 μM) and M1-Ub_4 (1 μM) were incubated with GST-MIU or GST with indicated concentration in the presence of dsDNA_20/23nt (2.7 μM) for 0.5h at 30°C. The reaction was analysed by SDS-PAGE.

Supplementary Figure 4. SPRTN core interacts with I44 patch of Ub in an avidity manner

(A) Isothermal titration calorimetry (ITC) analysis of the binding between SPRTN core (E112Q) and monoUb, SPRTN ZBD-BR and Ubs (monoUb, M1-Ub_2, M1-Ub_4). Details can also be found in Table S1.

(B) PAE plot of the ColabFold2 prediction of the interaction between SPRTN core and M1-Ub_2.

(C) MST analysis of the affinity between SPRTN core (E112Q) and monoUb. The dissociation constant (Kd) is indicated.

(D) Isothermal titration calorimetry (ITC) analysis of the binding between SPRTN core (E112Q) and M1-Ub_2 variants (I44A, I120A, I44AI120A). Details can be also found in Table S1.

(E) Validation of M1-Ub_2 variants on the effect of SPRTN activation. Recombinant full-length SPRTN (2 μM) and H1-C-Cy5 (1 μM) were incubated in the presence of dsDNA_20/23nt (2.7 μM) in combination with M1-Ub_2 variants (WT, I44A, I120A, I44AI120A, all at 2 μM) with the indicated time at 30°C. The reaction was analysed with SDS-PAGE followed by Cy5-scanning on Typhoon FLA 9500 (GE Healthcare) and Instant Blue staining. Representative figure from 3 repeats.

(F) Kinetics of the full-length H1-C-Cy5 substrate (C-terminal cleavage rate) and the full-length SPRTN (auto-cleavage rate) from Supplementary Figure 4E. Cy5 signals were analysed by ImageJ. SPRTN-FL signals were analysed by the iBright Analysis Software (Invitrogen). Kinetic data were fitted with one phase exponential decay - least squares fit (Prism). N=3. Error bar, SD.

Supplementary Figure 5. SPRTN core rapidly resolves polyubiquitinated DPCs

(A) SPRTN core cleavage assay towards Cy5-N-H1 and M1-Ub_4-Cy5-N-H1. Recombinant SPRTN core (2 μM) and H1 substrates (1 μM) were incubated in the presence of dsDNA_20/23nt (2.7 μM) with the indicated time at 30°C. Representative figure from 3 repeats.

(B) Kinetics of the full-length H1 substrates (N-terminal cleavage rate) from Supplementary Figure 5A. Kinetic data for H1 were fitted with simple linear regression. Kinetic data for Ub_4-H1 were fitted with one phase exponential decay - least squares fit (Prism). n=3. Error bar, SD.

(C) Activity of SPRTN core mutants towards M1-Ub_4-Cy5-N-H1. Recombinant SPRTN core variants (2 μM) and M1-Ub_4-Cy5-N-H1 (1 μM) were incubated in the presence of dsDNA_20/23nt (2.7 μM) with the indicated time at 30°C. Representative figure from 3 repeats.

(D) Kinetics of the full-length M1-Ub_4-Cy5-N-H1 (N-terminal cleavage rate) from Supplementary Figure 5C. Kinetic data were fitted with one phase exponential decay - least squares fit (Prism). n=3. Error bar, SD.
(E) Western blot analysis of the SPRTN core cleavage assay towards the H1 substrates. Recombinant SPRTN core (2 μM) and H1 substrates (1 μM) were incubated in the presence of dsDNA_20/23nt (2.7 μM) with the indicated time at 30°C. The reactions were analysed by SDS-PAGE followed by Cy5-scanning and then applied to western blot by Anti-His antibody on an iBright 1500 imaging system (Invitrogen). All the three H1 substrates are His-tagged on the N-terminal.
(F) SPRTN core cleavage assay towards the defined H1 substrates from Figure 6A. Recombinant SPRTN core (2 μM) and H1 substrates (1 μM) were incubated in the presence of dsDNA_20/23nt (2.7 μM) with the indicated time at 30°C. Representative figure from 3 repeats.
All the reactions were analysed by SDS-PAGE followed by Cy5-scanning on an iBright 1500 imaging system (Invitrogen) and Instant Blue staining. Cy5 signals from Supplementary Figure 5A and S5E were analysed by the iBright Analysis Software (Invitrogen).

Supplementary Figure 6. SPRTN UBZ and SPRTN core work in concert to resolve polyubiquitinated DPC substrates
(A) Isothermal titration calorimetry (ITC) analysis of the binding between SPRTN UBZ (or UBZ* domain) and Ubs (monoUb, M1-Ub_2 variants). The dissociation constant (Kd) and stoichiometry of binding (N) are indicated. Details can also be found in Table S3.
(B) SEC-MALS analysis of SPRTN UBZ and UBZ* domain. Parameters are listed in the Table S4. LS: light scattering; RI: refractive Index.
(C) Ub-binding comparison between SPRTN UBZ and SPRTN core. Complex models are predicted by AlphaFold3. monoUb and the distal Ub from M1-Ub_2 are imposed in the same orientation.
(D) Peak volume ratio of bound/free states for individual residues from Figure 7B. Absolute peak volume was quantified by the MestReNova software. Peaks that are completely broadened upon the addition of UBZ to monoUb have a peak volume ratio of 0.
(E) Surface plasmon resonance (SPR) analysis of the interaction between SPRTN PIP-UBZ and monoUb/PCNA/monoUb-PCNA. The dissociation constant (Kd) of binding is indicated here and listed in Table S5.
(F) Western blot analysis of the SPRTN cleavage assay towards PCNA or monoUb-PCNA. Recombinant full-length SPRTN (2 μM) and substrates (PCNA or monoUb-PCNA, 1 μM) were incubated in the presence of dsDNA_20/23nt (2.7 μM) with the indicated time at 30°C. Representative figure from 4 repeats. The reactions were analysed by SDS-PAGE and western blot with Anti-PCNA antibody, followed by re-blot with Anti-SPRTN antibody on an iBright 1500 imaging system (Invitrogen). Right panel: Quantification of the cleavage of PCNA and monoUb-PCNA. Band signals were quantified by the iBright Analysis Software (Invitrogen) and visualized by Prism. Significant analysis was performed by comparing 2h and 4h data points to 0h within

each condition by unpaired t-test (Prism). n=4. Error bar, SD. *p <0.05; **p <0.005; ***p <0.0005; ****p <0.00005; ns: not significant.

(G) Predicted SPRTN-FL structure (AlphaFold Protein Structure Database Entry: AF-Q9H040-F1-v4). The unstructured region (G222-K452) is coloured in cyan. The N-terminal MIU domain is coloured in orange. The C-terminal UBZ domain is colored in magenta.

Supplementary Tables

Table S1[a]: Affinity summary of SPRTN core (E112Q) with ubiquitin from ITC (Unit: μM)

		monoUb	M1-Ub_2				M1-Ub_4	M1-Ub_6
**variant**		**WT**	**WT**	**I44A**	**I120A**	**I44AI120A**	**WT**	**WT**
**SPRTN core (E112Q)**	Kd	undetectable	132.0 ± 3.9	undetectable	undetectable	undetectable	69.7 ± 6.9	34.8 ± 3.8
	N	N/A	0.085 ± 0.002	N/A	N/A	N/A	0.090 ± 0.012	0.128 ± 0.004
**SPRTN ZBD-BR**	Kd	undetectable	undetectable	-	-	-	undetectable	undetectable

a. Data from each ITC titration pair were collected from at least 3 repeats, except for the undetectable data with only 1 repeat. "-": not determined.

Table S2[a]: Affinity summary of ssDNA (20 nt) with SPRTN core (E112Q) in the presence of ubiquitin from ITC (Unit: μM)

		SPRTN core E112Q	SPRTN core E112Q + monoUb	SPRTN core E112Q + M1-Ub_4
**ssDNA (20nt)**	Kd	4.33 ± 0.98	5.15 ± 1.36	0.44 ± 0.03
	N	0.36 ± 0.05	0.36 ± 0.02	0.29 ± 0.01

a. Data from each ITC titration pair were collected from at least 3 repeats.

Table S3[a]: Affinity summary of SPRTN UBZ with ubiquitin from ITC (Unit: μM)

UBZ Variant		monoUb	M1-Ub_2	M1-Ub_2-I44A	M1-Ub_2-I44AI120A	M1-Ub_4
**WT**	Kd	1.58 ± 0.03	1.61 ± 0.23	1.67 ± 0.14	undetectable	1.55 ± 0.05
	N	0.73 ± 0.03	0.39 ± 0.01	0.86 ± 0.03	N/A	0.21 ± 0.01
**C456A, C459A**		undetectable	undetectable	-	-	-

a. Data from each ITC titration pair were collected from at least 3 repeats, except for the undetectable data with only 1 repeat. "-": not determined.

Table S4: Parameters of SPRTN UBZ and UBZ* from SEC-MALS

	His-UBZ	His-UBZ*
**Mn[a] (kDa)**	6.7 (± 5.5%)	6.6 (± 5.9%)
**Mw[b] (kDa)**	6.8 (± 5.5%)	6.7 (± 6.1%)
**Polydispersity (Mw/Mn)**	1.003 (± 7.7%)	1.014 (± 8.5%)

a. Mn: numeric-average molar mass; b. Mw: mass-average molar mass.

Table S5: Affinity summary of SPRTN PIP-UBZ with monoUb/PCNA/monoUb-PCNA from SPR (Unit: μM)

		monoUb	PCNA	monoUb-PCNA
**PIP-UBZ**	Kd	4.2 ± 0.9	12.8 ± 2.0	7.9 ± 1.2

KEY RESOURCES TABLE

REAGENT or RESOURCE	SOURCE	IDENTIFIER
**Antibodies**		
Mouse monoclonal anti-dsDNA	Abcam	Cat#ab27156
Mouse monoclonal anti-His	Novagen	Cat#70796-3
Mouse monoclonal anti-PCNA	Santa Cruz	Cat#SC-056
Rabbit polyclonal anti-H1.0	Proteintech	Cat#17510-1-AP
Rabbit polyclonal anti-SPRTN	Atlas	Cat#HPA025073
**Bacterial and virus Strains**		
BL21(DE3) Competent *E. coli*	NEB	Cat#C2527I
Rosetta™(DE3) Competent Cells	Millipore	Cat#70954
**Chemicals, peptides, and recombinant proteins**		
1, 10-Phenanthroline	Sigma	Cat#131377
250 kDa Plus Prestained Protein Marker	Vazyme	Cat#MP202-01
Benzonase	Millipore	Cat#71205
cOmplete, EDTA-free, Protease inhibitor cocktail tablets	Roche	Cat#05056489001
Cy5 Maleimide Mono-Reactive Dye	Cytiva	Cat#PA25031
Di-ubiquitin explorer panel	UbiQ	Cat#UbiQ-L01
DL2000 Plus DNA Marker	Vazyme	Cat#MD101-01
DL15000 Plus DNA Marker	Vazyme	Cat#MD103-01
DTT	Fluorochem	Cat#M02712
GSH (reduced form)	SERVA	Cat#23150.04
IGEPAL	Millipore	Cat#1.08603.6025
IPTG	UBPBio	Cat#P1010-10
ISG15	Abcam	Cat#ab268685
Lumitein™ Protein Gel Stain, 100X	Biotium	Cat#21002-1
gibco® MEM vitamin solution, 100X	Thermo Fisher	Cat#11120052
MIU peptide	GenScript	Custom synthesized
MOPS SDS Running Buffer, 20X	Invitrogen	Cat#NP0001
^{15}N-NH_4Cl	Sigma	Cat#609471-10G
NuPAGE® LDS Sample Buffer, 4X	Invitrogen	Cat#NP0007
Pefabloc SC (AEBSF)	Roche	Cat#11429868001
Phanta Max Master Mix (Dye Plus), 2X	Vazyme	Cat#P525-01
Phusion™ Hot Start II DNA Polymerase	Thermo Fisher	Cat#F549L
Precision Plus Protein™ Dual Color Standards, 10-250 kDa	Bio-Rad	Cat#1610374
Precision Plus Protein™ Dual Xtra Prestained Protein Standards, 2-250 kDa	Bio-Rad	Cat#1610377
Q5® High-Fidelity Master Mix, 2X	NEB	Cat#M0492S
QuickBlue Protein Stain	LubioScience	Cat#LU001000-LBO-1L
Rapid Taq Master Mix, 2X	Vazyme	Cat#P222-01
SMCC	Thermo Fisher	Cat#22360

SUMO1	R&D	Cat#UL-715-500
SUMO2	R&D	Cat#UL-753-500
SUMO3	UBPBio	Cat#E3300
SUMO2 chains (2-7)	ENZO	Cat#BML-UW9670
TCEP hydrochloride	APExBIO	Cat#B6055
Ubiquitin from bovine erythrocytes	Sigma	Cat#U6253-25MG
**Critical commercial assays**		
ClonExpress Ultra One Step Cloning Kit	Vazyme	Cat#C115-01
QuikChange Lightning MultiSite-Directed Mutagenesis Kit	Agilent	Cat#210515
NEBuilder® HiFi DNA Assembly Cloning Kit	NEB	Cat#E5520S
Amine Coupling Kit	Cytiva	Cat#BR100050
**Recombinant DNA**		
pGEX-6P1-hUb	Stiegliz Lab	N/A
pNIC28-Ub	This study	N/A
pET-47b-N-His-Ub_2	Stiegliz Lab	N/A
pET-47b-N-His-Ub_2, I44A	This study	N/A
pET-47b-N-His-Ub_2, I120A	This study	N/A
pET-47b-N-His-Ub_2, I44AI120A	This study	N/A
pET-24-tetraUb	Stiegliz Lab	N/A
pET-24-hexaUb	This study	N/A
pET-24-hexaUb, I44AI120A	This study	N/A
pGEX2TK-NEDD8	Stiegliz Lab	N/A
pET-22b-2xStrep-TEV-SPRTN	This study	N/A
pGEX-4T1-SPRTN-MIU (1-25)	This study	N/A
pGEX-4T1-SPRTN-ΔC (Lys241AsnfsX8)	This study	N/A
pNIC28-SPRTN-UBZ (452-489)	This study	N/A
pNIC28-SPRTN-UBZ (452-489), C456AC459A	This study	N/A
pNIC28-SPRTN core (26-240)	This study	N/A
pNIC28-SPRTN core (26-240), E112Q	This study	N/A
pNIC28-SPRTN core (26-240), L38A	This study	N/A
pNIC28-SPRTN core (26-240), L99A	This study	N/A
pGEX-6P1-SPRTN-ZBD-BR (151-245)	This study	N/A
pCold I-SPRTN-PIP-UBZ (320-489)	This study	N/A
pCold I-mH1-Ccys (S192C)	This study	N/A
pCold I-mH1-Ncys (M1-C-T2)	This study	N/A
pCold I-Ub-mH1-Ncys (M1-C-T2)	This study	N/A
pCold I-Ub_4-mH1-Ncys (M1-C-T2)	This study	N/A
pCold I-2xISG15-C78S-mH1-Ncys (M1-C-T2)	This study	N/A
pET28-mUbe1	Stiegliz Lab	N/A
pGEX6P1-UbcH5c, S22R	This study	N/A
pET16b-PCNA	Addgene	#134898
**Software and algorithms**		

ColabFold	Google	https://colab.research.google.com/github/sokrypton/ColabFold/blob/main/AlphaFold2.ipynb#scrollTo=G4yBrceuFbf3
ImageJ	NIH	https://imagej.net/software/fiji/downloads
Image Lab	Bio-rad	https://www.bio-rad.com/en-uk/product/image-lab-software?ID=KRE6P5E8Z
MestReNova	Mestrelab	https://mestrelab.com/download
Prism	GraphPad	https://www.graphpad.com
Topspin	Bruker	https://www.bruker.com/en/products-and-solutions/mr/nmr-software.html
**Other**		
Amicon® Ultra - 0.5 mL Centrifugal Filters - 3K	Millipore	Cat#UFC500324
Amicon® Ultra - 0.5 mL Centrifugal Filters - 10K	Millipore	Cat#UFC501024
Amicon® Ultra - 15 Centrifugal Filters - 3K	Millipore	Cat#UFC900324
Amicon® Ultra - 15 Centrifugal Filters - 10K	Millipore	Cat#UFC901024
Amicon® Ultra - 15 Centrifugal Filters - 30K	Millipore	Cat#UFC903024
Dialysis tube, 8 KDa cut-off	Cytiva	Cat#28955965
FastPure Plasmid Mini Kit	Vazyme	Cat#dc201-01
Glutathione Sepharose High Performance, 25 mL	Cytiva	Cat#17527901
HiLoad™ 16/600 Superdex™ 75 pg	Cytiva	Cat#28989333
HiPrep Heparin Fast Flow 16/10	Cytiva	Cat#28-9365-49
HisTrap™ FF, 5 mL	Cytiva	Cat#17531901
HiTrap SP HP, 5 mL	Cytiva	Cat#17115101
Mini Dialysis Kit, 1kDA cut-off, 250 uL	Cytiva	Cat#80-6483-75
Mini-PROTEAN® TGX™ Precast Protein Gels, 4-20%, 15-Well, 15 uL	Bio-Rad	Cat#4561096
Monolith® NT.115 series Capillaries	Nanotemper	Cat#MO-K022
Ni-NTA Agarose, 25 mL	Invitrogen	Cat#60-0442
Novex™ 4-20% Tris-Glycine Plus Midi Gels, 26-well	ThermoFisher	Cat#WXP42026BOX
Q Sepharose Fast Flow, 25 mL	Cytiva	Cat#17051010
Resource S, 1 mL	Cytiva	Cat#17117801
Series S Sensor Chip CM5	Cytiva	Cat#BR1000530
Slide-A-Lyzer™ Dialysis cassette, 3.5K MWCO	ThermoFisher	Cat#66330

Superose® 6 Increase 10/300 GL	Cytiva	Cat#29091596
Superdex™ 200 Increase 10/300 GL	Cytiva	Cat#28-9909-44
Zeba™ Spin Desalting Columns, 7K MWCO, 2 mL	ThermoFisher	Cat#89890

**OLIGONUCLEOTIDE**[a]	**SEQUENCE (5' to 3')**
FAM-Turner-20bp-F	FAM-ACGCCTGAAGAGTCTGGTGA
Amide-WS-23bp-R	Amine-TCACCAGACTCTTCAGGCGTtcc
15nt-dA (ssDNA_dA_15nt)	AAAAAAAAAAAAAAA
15nt-dT (ssDNA_dT_15nt)	TTTTTTTTTTTTTTT
Turner-20bp-F (ssDNA_20nt)	ACGCCTGAAGAGTCTGGTGA
Turner-20bp-R	TGCGGACTTCTCAGACCACT
WS-23bp-F (ssDNA_23nt)	GGAACGCCTGAAGAGTCTGGTGA
WS-23bp-R	TCACCAGACTCTTCAGGCGTtcc
OD4_Fw (ssDNA_100nt)	ACGCGGGTTAGCGGTACCCAGTCCAGTGACCTAGGCA GCTTTAAGCTAGTACGACTTGCTTAGATTGCAGTCGAC GACGTAGCTGGCATAGAGGTACAGC
OD4_Rev	GCTGTACCTCTATGCCAGCTACGTCGTCGACTGCAATC TAAGCAAGTCGTACTAGCTTAAAGCTGCCTAGGTCACT GGACTGGGTACCGCTAACCCGCGT
WS-10bp-R-nick1	TCACCAGACT
WS-10bp-R-nick2	CTTCAGGCG
WS-23bp-fork-F	CCCCTTTTGAGGAACGCCTGAAGAGTCTGGTGA
WS-23bp-fork-R	CCCCTTTTGACCTTGCGGACTTCTCAGACCACT

Source: all synthesised from Invitrogen except FAM-Turner-20bp-F from Sigma
